# MXene Contact Engineering for Printed Electronics

**DOI:** 10.1002/advs.202207174

**Published:** 2023-04-25

**Authors:** Zhiyun Wu, Shuiren Liu, Zijuan Hao, Xuying Liu

**Affiliations:** ^1^ School of Materials Science and Engineering Zhengzhou Key Laboratory of Flexible Electronic Materials and Thin‐Film Technologies Zhengzhou University Zhengzhou 450001 P. R. China; ^2^ Henan Innovation Center for Functional Polymer Membrane Materials Xinxiang 453000 P. R. China

**Keywords:** electrical performance, energy levels, MXene contact, printed electronics, printing technologies

## Abstract

MXenes emerging as an amazing class of 2D layered materials, have drawn great attention in the past decade. Recent progress suggest that MXene‐based materials have been widely explored as conductive electrodes for printed electronics, including electronic and optoelectronic devices, sensors, and energy storage systems. Here, the critical factors impacting device performance are comprehensively interpreted from the viewpoint of contact engineering, thereby giving a deep understanding of surface microstructures, contact defects, and energy level matching as well as their interaction principles. This review also summarizes the existing challenges of MXene inks and the related printing techniques, aiming at inspiring researchers to develop novel large‐area and high‐resolution printing integration methods. Moreover, to effectually tune the states of contact interface and meet the urgent demands of printed electronics, the significance of MXene contact engineering in reducing defects, matching energy levels, and regulating performance is highlighted. Finally, the printed electronics constructed by the collaborative combination of the printing process and contact engineering are discussed.

## Graphical abstract

From a viewpoint of the contact engineering, this review summarizes the critical factors impacting device performance in MXene‐based printed electronics. The significance of MXene contact engineering in reducing interfacial defects, matching energy levels, and regulating performance is highlighted. The application scenarios of MXene contact engineering in printing electronics and the current challenges and future perspectives are discussed.

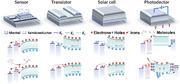

## 1 Introduction

Modern technology for silicon‐based integrated circuit involves hundreds of steps including preparing monocrystalline silicon substrates, manufacturing billions of transistors, and interconnecting them together. In contrast to that, printed electronics can be additively manufactured, normally constructed by stacking electronic components through printing technology to form electronic layers and junction interfaces for charge carrier separation, storage, or transportation.^[^ ^1^ ^]^ During this process, the manufacturing technology for printed electronics is similar to the traditional printing process except that the used “ink” is an electronic material with conductive, dielectric, or semiconductor properties. In most of the reported results, the electrical performance of printed electronic devices is strongly correlated with thin‐film morphology,^[^ ^2^ ^]^ the work function of metal electrodes,^[^ ^3^ ^]^ and the interfacial state sandwiched by metal and semiconductor,^[^ ^4^ ^]^ which in a broader sense are considered as the impact factors of the contact engineering in the semiconductor device. Therefore, in recent years, researchers proposed a wide variety of strategies to tune the contact state so that the performance of printed electronics can be optimized, including chemical or physical modification of metal electrodes or semiconductors, and introduction of a charge injection layer.^[^ ^5^ ^]^ Until now, a large number of scientific papers have been published to review the investigation of using tunable contact state to enhance the device performance from the aspects of semiconductors, while few tend to discuss the contribution of tunable metal contact in this field.

**Figure 1:**
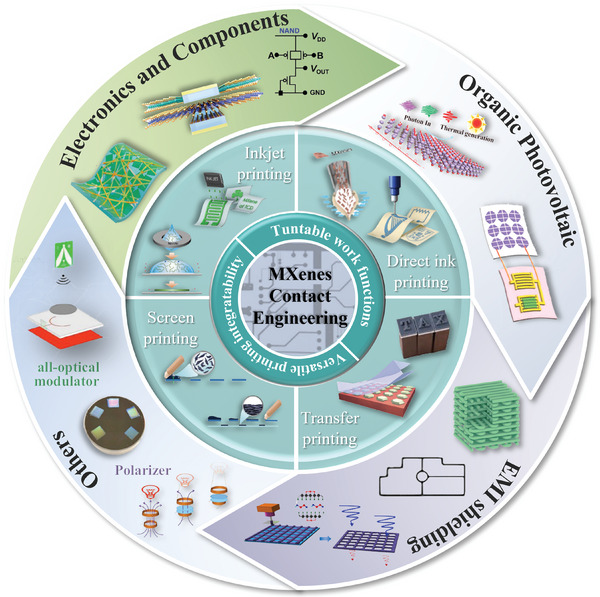
Techniques, principles, and applications of MXene contact for printed electronics. The printing processes include inkjet printing: Reproduced with permission.^[^ ^17^ ^]^ Copyright 2019, Springer Nature. Direct ink printing: (left) Reproduced with permission.^[^ ^10^ ^b]^ Copyright 2021, Wiley‐VCH. (right) Reproduced with permission.^[^ ^17^ ^]^ Copyright 2019, Springer Nature. Screen printing: Reproduced with permission.^[^ ^18^ ^]^ Copyright 2021, Wiley‐VCH. Transfer printing: (left) Reproduced with permission.^[^ ^19^ ^]^ Copyright 2018, Springer Nature. (right) Reproduced with permission.^[^ ^9a^ ^]^ Copyright 2021, American Chemical Society. Application scenarios include electronics and components: (from left to right) Reproduced with permission.^[^ ^20a^ ^]^ Copyright 2021, Elsevier. Reproduced with permission.^[^ ^20b^ ^]^ Copyright 2019, American Chemical Society. Reproduced with permission.^[^ ^10b^ ^]^ Copyright 2021, Wiley‐VCH. Organic photovoltaic. Reproduced with permission.^[^ ^21^ ^b]^ Copyright 2020, American Chemical Society. (bottom) Reproduced with permission.^[^ ^21a^ ^]^ Copyright 2021, Wiley‐VCH. EMI shielding: (left an middle) Reproduced with permission.^[^ ^16a^ ^]^ Copyright 2021, Elsevier. (right) Reproduced with permission.^[^ ^22^ ^]^ Copyright 2021, Springer Nature. Others: (from left to right) Reproduced with permission.^[^ ^23c^ ^]^ Copyright 2022, Elsevier. Reproduced with permission.^[^ ^23b^ ^]^ Copyright 2019, Springer Nature. Reproduced with permission.^[^ ^23a^ ^]^ Copyright 2022, American Chemical Society.

MXene electrodes with tunable electric properties have been widely employed as the contact in printed electronic devices because of their 2D structure with the chemical formula M*_n_*_+1_X*_n_* T*_x_*, where “M” represents an early transition metal (Sc, Ti, V, Cr, Zr, Nb, Mo, Hf, and Ta), “X” represents C, N, or C—N, and “T*_x_*” represents the surface terminals of O, F, and OH.^[^ ^6^ ^]^ MXene has high conductivity, high hydrophilicity, and large specific surface area.^[^ ^7^ ^]^ Moreover, MXene‐based ink has adjustable rheological properties, mechanical properties, electromagnetic wave absorption characteristics, photoelectric conversion and photothermal conversion ability.^[^ ^8^ ^]^ For printing electronic devices with MXene contact, the microstructure in or between thin films was also found to be tunable and responsible for promoting device performance.^[^ ^9^ ^]^ The barrier height trade‐off formed at the metal MXene/semiconductor contact interface was intensively reported in order to realize barrier‐free injection of carriers for controlling charge transfer and capacitive characteristics.^[^ ^10^ ^]^ In addition, the rich combination of elements in the MXene family, the spin–orbit coupling (SOC) effect, and dipole effect arising from the surface termination also contribute to tuning the engineering of MXene contact. As a result, the ideal metal/semiconductor interface assembled with versatile printing techniques has allowed the MXene ink for applying in energy storage,^[^ ^8^, ^11^ ^]^ sensing,^[^ ^12^ ^]^ actuation,^[^ ^13^ ^]^ transistors,^[^ ^10^, ^14^ ^]^ photovoltaic,^[^ ^15^ ^]^ electromagnetic shielding,^[^ ^16^ ^]^ and other fields, as summarized in **Figure** **1**. Thus, taking the importance of MXene contact into consideration, a comprehensive review regarding methodology and principle will be necessary for abroad interest in printed electronics.

This review begins with a summary of the synthesis strategies, dispersion, and printing processes of MXene inks. Second, the basic electrical properties of MXenes are described regarding the aspects of calculation and simulation, electrical properties, the tunability of energy band structure, and contact resistance caused by surface functionalization. Of note, the contact principle and functional adjustability of MXenes are summarized and discussed with respect to the contact barrier, the pinning effect, work function, and heterogeneous contact of MXene‐based electronics. Finally, the application scenarios of MXene contact engineering in printing electronics in the past five years are discussed, and the current challenges and future perspectives of MXene‐based printed electronics are proposed.

## 2 Preparation and Printing Techniques of MXene Inks

### 2.1 Synthesis Strategies

The chemical and physical characteristics (like surface dipole moment, surface potential, and work function) of MXene contact have been investigated to strongly depend on their synthetic methods. In general, the synthetic methodologies of 2D layered MXenes can be divided into two distinct routes: i) the top‐down approach and ii) the bottom‐up method. The top‐down approach is always achieved by etching the A element (“A” represents a group IIIA or VIIIA element) from layered ternary MAX precursors, followed by exfoliation into nanosheets (NSs), and both steps are conducted in dispersions. Selective chemical etching is the most commonly used method to prepare MXenes, and the obtained products are permanently terminated by —O, —OH, —F, or more likely, a combination of all species.^[^ ^24^ ^]^ The abundant functional groups and high negative charge (zeta potential is about −30 mV) on its surface are responsible for stable colloidal dispersion in water or various organic liquids, which is essential for forming printing ink and tunning surface charge distribution.^[^ ^25^ ^]^ The bottom‐up method is typically realized by organic/inorganic/atomic synthesis or deposition, e.g., chemical vapor deposition,^[^ ^26^ ^]^ salt template method,^[^ ^27^ ^]^ and plasma‐enhanced deposition.^[^ ^28^ ^]^ Compared with the etching synthesis route, higher temperature and pressure are needed during the bottom‐up process; the obtained flake size is relatively larger without —O and —OH terminals, which prefers to be used in the high‐precision fabrication of nanosize contacts.^[^ ^29^ ^]^ Therefore, MXenes prepared by the etching method are more suitable for printing electronic devices. Herein, we will focus on the introduction of wet chemical etching in the following discussion.

#### 2.1.1 Hydrofluoric Acid (HF) Etching

HF etching is the first reported approach to successfully fabricate Ti_3_C_2_T*_x_* MXenes by selective chemical etching of MAX phases.^[^ ^30^ ^]^ Specifically, the metal M—A bond is more reactive than the metal/ion/covalent M—X bond, so the A— element can be removed without destroying M*_n_*_+1_X*_n_* layered structures by HF due to its low binding energy. During the etching process, the A— element reacts with HF to form fluorides (AlF_3_, SiF_4_), gaseous hydrogen (H_2_), and accordion‐like M*_n_*_+1_X*_n_*. Meanwhile, the polycrystalline MXenes were etched into single crystals, resulting in a high etch yield and minimizing the amount of residual MAX phase.^[^ ^24^ ^]^ The etching reaction can be simply described by the following Equations (1)–(3)

(1) $$\begin{array}{ccc} & & M_{n+1}{\mathrm{AX}}_{n}+3\mathrm{HF}\left(\mathrm{or}\;4\mathrm{HF}\right)\to M_{n+1}X_{n}+{\mathrm{AF}}_{3}\left(\mathrm{or}\;{\mathrm{AF}}_{4}\right)\\ & & +\;3/2H_{2}\left(\mathrm{or}\;2H_{2}\right) \end{array}$$

(2) $$M_{n+1}X_{n}+2H_{2}O\to M_{n+1}X_{n}{\left(\mathrm{OH}\right)}_{2}+H_{2}$$

(3) $$M_{n+1}X_{n}+2\mathrm{HF}\to M_{n+1}X_{n}F_{2}+H_{2}$$

It should be noted that each MAX has different stability and reactivity, relative to different etchants. The optimal etching conditions for different MAX phases depend on the atomic bonding strength, particle size, and crystal structure. In general, transition metals with greater atomic numbers require longer etching times and stronger etchants. More than 30 kinds of MXenes have been synthesized with HF.^[^ ^31^ ^]^ The surface chemistry and structural properties of MXenes can be tuned by adjusting the etching concentration, allowing for alternative surface groups and variable surface dipole moment that are correlated with the work functions of functional materials.^[^ ^11^ ^]^ For instance, different HF concentrations lead to different surface functions (—OH, —O, and/or —F), densities defect, and different work functions. A low concentration of HF results in a higher oxygen/fluorine ratio terminated on the MXene surface, while a higher concentration of HF solution leads to stronger interactions and more defects in the resulting product.^[^ ^24^ ^]^

Selective etching with HF is also suitable for bimetallic MXene precursors, such as Mo_2_TiC_2_T*_x_*, Mo_2_Ti_2_C_3_T*_x_*, and Cr_2_TiC_2_T*_x_*, which can be etched in 48–51% HF solution. Recently, Syamsai et al.^[^ ^32^ ^]^ successfully prepared Ti*_x_* Ta_(4−_ *_x_*_)_C_3_ via the above etching strategy. When Ta and Ti form an alloy compound, Ta atoms and Ti atoms occupy the outer layer and the intermediate layer, respectively, thereby easing up the self‐oxidation problem of tantalum. Although more than ten “A” elements have been reported in the MAX phase precursor, so far, only Al and Si have been successfully etched into MXene. Furthermore, the M_2_AX phase requires milder etching conditions than the M_3_AX_2_ and M_4_AX_3_ phases.^[^ ^33^ ^]^

#### 2.1.2 Fluoride‐Based Salt Etching

The direct use of HF as acids is fraught with unpredictable risks and the synthetic yields are frequently below 20%.^[^ ^34^ ^]^ In the following contexts, several mild etching strategies developed by in situ generations of HF through mixing alkali metal fluoride (LiF,^[^ ^35^ ^]^ KF^[^ ^36^ ^]^) with hydrochloric acid (HCl) will be discussed. Since the spontaneous insertion of Li^+^ into the multiple particles and their surface proton exchange further increased the interlayer spacing, the interaction between the adjacent MXene sheets is significantly weakened. Eventually, the MXene flakes were delaminated into a clay structure upon solvent exchange at ease (i.e., washing and manual shaking). Typically, the above etching process of the strategy always begins from the outermost edge of MAX particles; such a mild etching environment does not destroy the grain boundary of MXenes, and is easy to leave the incomplete etched MAX particles at last. Compared with HF route, this route can produce MXenes with larger lateral sizes, fewer pinholes/defects, and higher O/F ratio. Ultrasonic treatment is often used to assist in the lamellar peeling of MXenes, but it can destroy the electrical conductivity and structural integrity of the flakes. The organic‐solvent‐assisted intercalation collection method for preparing of Ti_3_C_2_T*_x_* MXenes could alleviate the issues caused by intense ultrasonic treatment and high‐speed centrifugation. For example, intercalation with dimethylsulfoxide (DMSO) followed by the addition of dichloromethane to remove DMSO only requires low‐speed centrifugation during the post‐treatment and the yield reached 46% up to 70% after six cycles.^[^ ^37^ ^]^

#### 2.1.3 Fluorine‐Free Etching

**Figure 2:**
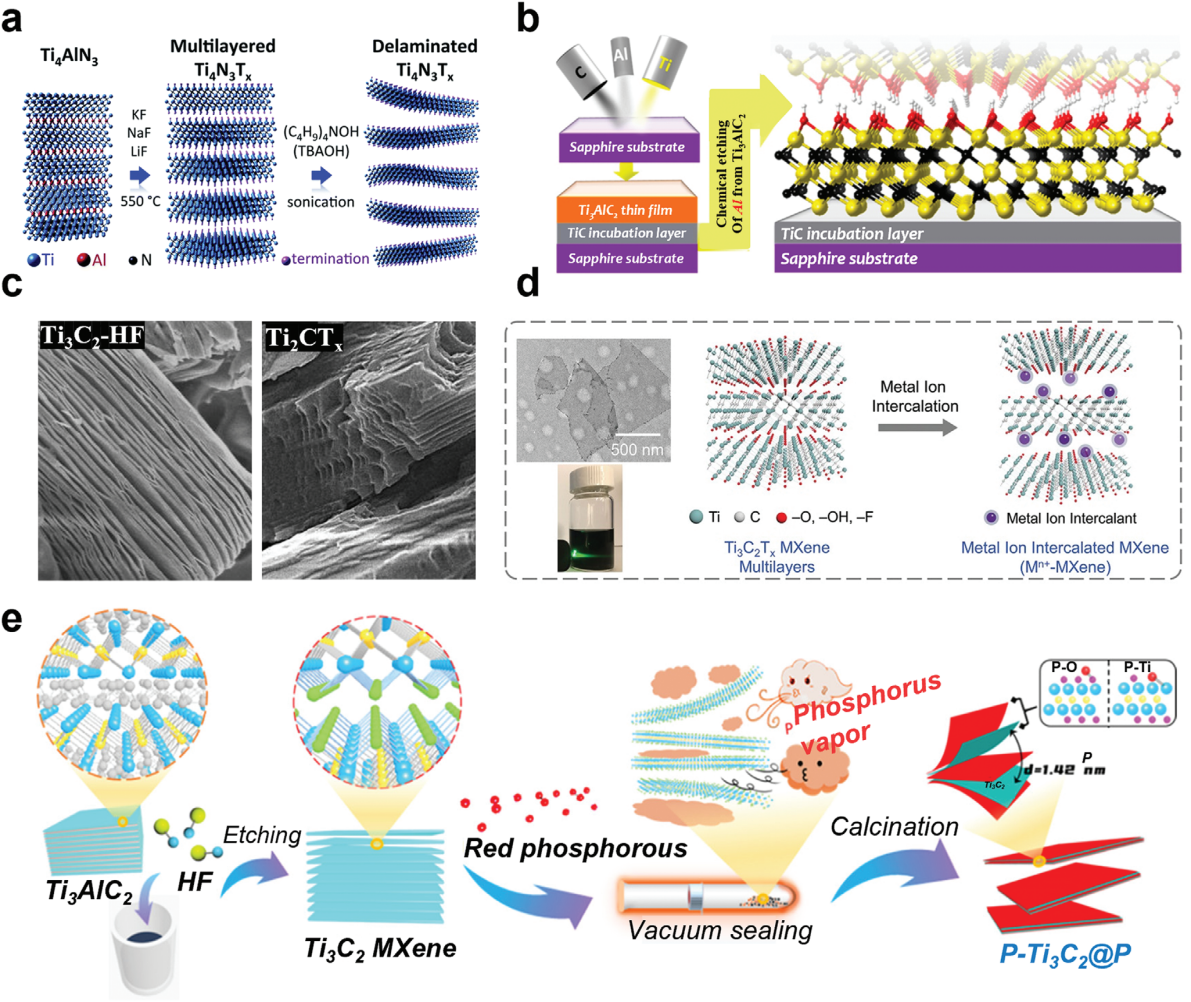
a) The schematic diagram illustrates the synthesis of Ti_4_N_3_T*_x_* from Ti_4_AlN_3_ by molten salt treatment at 550 °C under Ar condition, followed by the dispersion of multilayer MXenes using TBAOH. Reproduced with permission.^[^ ^41^ ^]^ Copyright 2016, Royal Society of Chemistry. b) The picture on the left show magnetron sputtering of Ti, Al, and C to form several nanosized TiC incubation layers on a (0001) sapphire substrate, followed by the deposition of Ti_3_AlC_2_. The right diagram is that of Ti_3_C_2_ at —OH terminal after Al selective etching by Ti_3_AlC_2_ (Ti atom is yellow, C atom is black, O atom is red, and H atom is white). Reproduced with permission.^[^ ^42^ ^]^ Copyright 2014, American Chemical Society. c) Scanning electronic microscopy (SEM) picture of MAX phase treated with standard HF (left), SEM picture of MAX phase etched with EMIMBF_4_ and BMIMPF_6_ (right). Reproduced with permission.^[^ ^44^ ^]^ Copyright 2020, Royal Scoiety of Chemistry. d) Schematic diagram of spontaneous insertion of metal ions Ca^2+^, Mg^2+^, and Al^3+^ into MXene electrode. Inset on the left is a TEM image of a stripped Ti_3_C_2_T*_x_* MXene nanosheet and the Tyndall scattering effect of the MXene dispersion. Reproduced with permission.^[^ ^46^ ^]^ Copyright 2020,Wiley‐VCH. e) Schematic of Ti_3_C_2_ MXene delamination and intercalation with phosphorus vapor. Reproduced with permission.^[^ ^63^ ^]^ Copyright 2021, Royal Scoiety of Chemistry.

Mainstream synthetic routes using HF or fluoride‐based compounds (LiF/HCl, ammonium bifluoride (NH_4_HF_2_), KHF_2_) and fluorine‐containing ionic liquids as etchants, which always incorporate the —F terminal, will degrade the performance of the resultant products and make surface modification and work function tunability hard.^[^ ^38^ ^]^ Therefore, fluorine‐free synthesis methods, such as alkali treatment, ammonium salt, ionic liquid–water mixture etching, or molten salt etching, have been developed. Alkali treatment adopts high temperature, high pressure, and high concentration of alkali‐assisted hydrothermal method to prepare MXenes Ti_3_C_2_T*_x_* (T = OH, O), where a number of —OH terminals are introduced and —F end groups are inhibited. The temperature and the concentration of the base solution are crucial factors concerning the generation and the relevant purity of the products.^[^ ^37^ ^]^ In electrochemical etching, Ti_3_AlC_2_ was used as the electrode, and ammonium chloride and tetramethylammonium hydroxide were used as the basic binary aqueous electrolyte (pH > 9), the current produces the protons needed for the acid to achieve the etching conditions, which can be used for the preparation of Ti_3_C_2_.^[^ ^39^ ^]^ The use of molten salt method can be traced back to 1999, when Ti_3_SiC_2_ was immersed in molten cryolite to etch the Si atoms to get Ti_3_C_2_.^[^ ^40^ ^]^ The first 2D metal nitride Ti_4_N_3_ was obtained by heating it with fluoride salt to etch aluminum from Ti_4_AlN_3_ powder precursor in argon atmosphere at 550 °C^[^ ^41^ ^]^ (**Figure** **2**a).

The synthesis of MXenes with aqueous NH_4_HF_2_ can effectively prevent the generation of harmful gases.^[^ ^42^ ^]^ This process starts with sputter deposition of Ti_3_AlC_2_ (initial formation of a TiC culture layer). Then, aluminum layer was etched by NH_4_HF_2_ to get a 2D Ti_3_AlC_2_ layer according to the Equations (4) and (5) and Figure 2b ^[^ ^42^ ^]^

(4) $${\mathrm{Ti}}_{3}{\mathrm{AlC}}_{2}+3{\mathrm{NH}}_{4}{\mathrm{HF}}_{2}={\left({\mathrm{NH}}_{4}\right)}_{3}{\mathrm{AlF}}_{6}+{\mathrm{Ti}}_{3}C_{2}+3/2H_{2}$$

(5) $${\mathrm{Ti}}_{3}C_{2}+{\mathrm{aNH}}_{4}{\mathrm{HF}}_{2}+{\mathrm{bH}}_{2}O={\left({\mathrm{NH}}_{3}\right)}_{c}{\left({\mathrm{NH}}_{4}\right)}_{d}{\mathrm{Ti}}_{3}C_{2}{\left(\mathrm{OH}\right)}_{x}F_{y}$$

However, the embedding of ammonium also introduced more H_2_O molecules into the MXene layer, resulting in much difficulty upon drying of the product. In this reaction system, although relatively large‐sized sheets can be obtained, a small amount of HF is still generated.^[^ ^43^ ^]^ In ionic liquid–water mixture etching route for Ti_3_AlC_2_ and Ti_2_AlC, F^−^ is present in the organic anions. Although this type of ion extended the etching time due to the relatively weak acidity, it is still advantageous over other HF‐free etching. The ionic liquid acts as both an etchant and an intercalator inducing a relatively large interlayer spacing. The morphology of the products is different from that of the accordion structure etched by HF, showing a cave‐like structure with smooth edges^[^ ^44^ ^]^ (Figure 2c).

Recently, Shen et al.^[^ ^45^ ^]^ adopted a green molten salt electrochemical method to synthesize fluorine‐free Ti_3_C_2_Cl_2_. Ti_3_AlC_2_ and Ni served as anode and cathode by adding the kinds of inorganic salts (Li_2_O and Li_2_S), different surface functional groups can be obtained, such as substituting —O or —S for —Cl.^[^ ^31^ ^]^ In addition, the fluorine‐free etching method can also be used to fabricate —Cl‐terminated MXenes (Ti_3_C_2_Cl_2_ and Ti_2_CCl_2_) in molten zinc oxide environment. The chloride‐terminated MXenes manifest excellent thermal stability, whereas the physical and chemical properties of these novel terminals are still awaiting further exploration^[^ ^46^ ^]^ (Figure 2d).

Because of the high chemical activity of Al, most of MXenes are continuously synthesized from the MAX phase containing Al. In addition, layered compounds composed of various transition metals such as Sc, Zr, Hf, in particular (MC)*_n_* [Al(A)]*_m_* C*_m_*_−1_, can also be applied as precursors to prepare MXenes. The relationship between the formation of layered carbides and the type of transition metal can be summarized based on the lattice mismatch between the cubic MC and Al_4_C_3_ cells.^[^ ^47^ ^]^ Specifically, compounds with a slight lattice mismatch preferentially form M*_n_*_+1_AlC*_n_* phases, and selective etching of these non‐MAX phases results in the corresponding MXene products. Zr_3_C_2_T*_x_*,^[^ ^48^ ^]^ Hf_3_C_2_T*_x_*,^[^ ^49^ ^]^ and Mo_2_C_9_^[^ ^50^ ^]^ have been successfully prepared using Zr_4_Al_3_C_5_, Hf_3_Al_4_C_6_ *_x_*, Mo_2_Ga_2_C as precursors, respectively. A novel 2D ScC*_x_* OH structure can also be achieved similarly to this method.^[^ ^51^ ^]^ These results indicate that MXenes can be synthesized without the MAX phase precursor.

### 2.2 Delamination of MXenes for Preparing of the MXene Inks

Delamination is necessary to obtain single‐ or few‐layer MXenes, which is a necessary step to explore their 2D state characteristics for electronic applications. Since M—A bond between MXenes is substituted by relatively weak van Waals and hydrogen bonds during the etch process, multilayer MXenes can be delaminated into single‐ or few‐layer. However, the strength of conventional mechanical exfoliation is not enough to break the interlayer force, so the yield is relatively low.^[^ ^52^ ^]^ Most single‐ or few‐layer MXene flakes are obtained via intercalation (such as cation intercalation, molecular intercalation) combined with physical delamination. Various organic, inorganic, and ionic substances can be intercalated between MXene layers to weaken the interactions between adjacent layers and increase the interlayer spacing (sonication). The most commonly used method is utilizing Li^+^ and HCl or NH_4_^+^ etchant to synthesize multilayer MXenes, followed by sonication to accomplish physical delamination. Since Li^+^ and NH_4_^+^ iron exist between MXenes layers, a MXene “clay” will be obtained.^[^ ^44^ ^]^ When mixed with fluoride salts and acids (such as HCl and LiF) as etchant, no additional molecules are required, because the etched product has been intercalated with metal cations.

Li^+^, Na^+^, K^+^, Cs^+^, and Mg^2+^ ions can also be embedded into Ti_3_C_2_ MXenes to realize successful delamination of MXenes.^[^ ^53^ ^]^ Chen et al.^[^ ^54^ ^]^ explored the effect of different ions on the properties of the resulting MXenes. Twelve metal ions, ranging from alkaline earth metal ions (Be^2+^, Mg^2+^, Ca^2+^), lanthanide metal ions (Gd^3+^, Tb^3+^, Ho^3+^), transition metal ions (Ag^+^, Cu^2+^, Mn^3+^, Cr^3+^, Fe^3+^), to post‐transition metal ions (Al^3+^, In^3+^), were intercalated to expand the nanofluidic channels of multilayered MXene electrodes, resulting in greatly facilitated charge‐transfer processes (Figure 2e). In addition, MXenes can be intercalated with various polar organic molecules, such as ethanol, DMSO,^[^ ^37^, ^55^ ^]^ dipicolinic acid,^[^ ^56^ ^]^ hydrazine, urea,^[^ ^57^ ^]^ isopropylamine,^[^ ^58^ ^]^ dodecyl dimethyl ammonium bromide (DDAB), aryl diazonium salts,^[^ ^59^ ^]^ followed by mechanical vibration or sonication in water, will lead to a colloidal solution of single or few‐layer MXenes. In addition to these polar solvents, large molecules such as organic bases, tetrabutylammonium hydroxide (TBAOH, (C_4_H_9_)_4_NOH), choline hydroxide, *n*‐butylamine, etc.,^[^ ^58^, ^60^ ^]^ can also be able to interact with multilayered MXenes, leading to a large increase in the interlayer spacing. Chen et al.^[^ ^9b^ ^]^ proposed a strategy based on holocellulose‐nanofibril (HCNF)‐assisted intercalation in achieving the high yield of MXenes. Upon the uniform mixing of multilayered MXenes and HCNF, as the expansion force of HCNF orientation and the expansion force of water destroy van der Waals (vdW) between layered nanosheets, the space between adjacent MXenes layers will become larger after several directional freezing and thawing cycles, and then high‐quality and large‐size layered MXene sheets are obtained by ultrasonic treatment.

The surface chemical properties of the material greatly affect the internal dispersion and the attractiveness of the liquid to the matrix. Therefore, understanding the surface chemistry of MXenes is essential for the dispersion and stability of ink and printing processes. According to the first principle, all surface‐terminated MXenes have negative formation energy and significant thermodynamic stability compared with bare MXene surfaces.^[^ ^61^ ^]^ As a result, MXenes synthesized by wet chemical methods usually have —OH, —O, and —F functional groups, derived from the F‐based wet etching processes. The abundant functional groups and high negative charge (zeta potential is about −30 mV) on its surface make it easy to form stable colloidal dispersion in various water‐based and organic solvents. For example, as a typical example of MXenes, Ti_3_C_2_T*_x_* has fantastic stability in aqueous dispersion systems, with a concentration window from less than 1 to hundreds of mg mL^−1^, without the use of any surfactants often needed in printable ink formulations.^[^ ^62^ ^]^ This is particularly essential for the preparation of inks because the residual surfactant may damage the electronic performance of the printed/coated film, so it needs to be removed by postprinting treatment (usually by heating), which limits the flexible substrate that can be used for printing. On the other hand, the stable dispersion and rheology of MXenes prevent the use of rheological modifiers, making it easier to adjust the rheology and concentration of MXene ink. Intuitively, the synthetic route adopted will affect the surface chemistry of MXenes, thus affecting its electronic functions or charge transporting behavior.

### 2.3 Printing of MXene Ink

In order to obtain and investigate the intrinsic properties of MXene materials, and to realize the fabrication of integrated devices with typical patterns and 3D structures, various printing/coating techniques like inkjet printing,^[^ ^21^, ^64^ ^]^ pen drawing,^[^ ^65^ ^]^ screen printing,^[^ ^9^, ^66^ ^]^spraying,^[^ ^2^, ^67^ ^]^ laser processing, suction filtration, and other assembly technologies of MXene materials have been employed to realize the pattern of MXenes.^[^ ^68^ ^]^ Among them, the printing is an effective and high‐precision patterning strategy. According to whether the contact happens between the substrate and printing apparatus, the processes are divided into noncontact printing such as 3D printing and inkjet printing, and the contact printing including screen printing and transfer printing. **Table** **1** summarizes the printing technology, viscosity, resolution, thickness, and application scenarios for different MXene‐based inks. These printing technologies differ in ink flow characteristics, resolution, film thickness, and scalability. In general, the viscosity requirements of the inks for the different printing processes are sequential (from high to low) as follows: 3D printing, screen printing, transfer printing, and inkjet printing. The printing thickness is in sequence as follows: 3D printing, screen printing, transfer printing, and inkjet printing. Some of them offer enormous possibilities for manufacturing of multilayered devices with multiple interfaces and contacts owing to their operation characteristics and the ability to realize large‐scale and low‐cost device fabrication.

**Table 1:** The printing technology, viscosity, print resolution, print thickness, and application scenarios of various MXene‐based inks. “MXene–LTO, MXene–LFP” represents MXene‐queous MXene‐based lithium titanate inks and MXene–lithium iron phosphate inks, respectively. “HCNF” represents holocellulose nanofibrils. “MXene–N” represents nitrogen‐doped MXene. “R–M–A” represents RuO_2_·*x*H_2_O nanoparticles, MXene, and AgNW ink. “MP” represents PH1000. “p‐MXene” represents the MXene ink with prepolymerized polydopamine‐macromolecule‐grafted surface. “GO–MXene–N” represents graphene oxide–nitrogen‐doped MXene. “Ti_3_C_2_@3DnCEs” represents MXene‐functionalized 3D‐printed nanocarbon electrodes. “PEO” represents polyethylene oxide. “10L” represents ten layers of print

Printing technology	Ink	Ink viscosity	Printing resolution [µm]	Printing thickness [µm]	Application	Ref.
Screen printing	MXene–LTO MXene–LFP	3548	–	2	Self‐powered integrated system	[18]
	HCNF/MXene	38.2	10000	100	Electromagnetic interference shielding	[9b]
	MXene sediment	35	≈235	1.4	Micro‐supercapacitors	[66]
	MXene–N	≈10^4^	–	–	–	[69]
	R–M–A ink	179	50	0.27	Micro‐supercapacitors	[9c]
	MXene	–	1000	–	Force sensors	[70]
Transfer printing	MXene	–	≈4000	3	Supercapacitors	[71]
Inkjet printing	MP hybrid ink	≈10^2^	30–160	0.25	Temperature sensor	[21a]
	MXene	5 × 10^3^	610	–	Conductive circuits	[62a]
	MXene	–	80	0.130	Supercapacitor	[72]
	Ti_3_C_2_T*_x_*/WSe_2_ nanohybrids	≈10^4^	–	0.06–0.18	Sensors	[73]
	MXene	2400	–	–	Photonic devices	[23b]
	p‐MXene	≈10^3^	100	2	Polarizer	
	MXene/DMSO	3400	120	1.35	Electromagnetic interference shielding	[74]
3D printing	GO–MXene–N	≈10^5^	300	15 000	Micro‐supercapacitors	[69]
	Ti_3_C_2_–MXene‐functionalized PEDOT:PSS	≈10^3^	–	295–600	Electromagnetic interference shielding	[16b]
	Ti_3_C_2_@3DnCEs	–	–	300	Capacitors	[75]
	Ti_3_C_2_T*_x_*/PEO	1.8 × 10^4^	–	3.2–12.1	Electromagnetic interference shielding	[16a]
	NiCoP/Ti_3_C_2_ MXene	≈10^4^	50	260	Supercapacitors	[76]
	MXene	≈10^3^	1–1000	1.5	Supercapacitors	[77]
3D freeze‐printing	Ti_3_C_2_T*_x_* aerogels	–	250	100	–	[78]
Direct ink writing	MXene/AlOOH	>10^4^	1000	750–2000	Electromagnetic interference shielding	[22]
	MXene	–	180 (10L)	0.15 (10L)	Logic circuits	[10b]
Extrusion printing	Ti_3_C_2_/PEDOT:PSS	>10^4^	250	15	Electromagnetic interference shielding	[79]

#### 2.3.1 Contact Printing

##### Screen Printing

As a typical contact printing, screen printing is a printing process using a patterned screen as a template, through which high–viscosity ink is squeezed onto substrate to realize patterned deposition. Typically, the rheological behavior of inks plays a crucial role in screen printing.^[^ ^80^ ^]^ Under specific shear stress conducted from a rubber squeegee, viscosity of the ink decreases linearly, allowing the flowing from the screen mesh to the surface of the substrate. When the shear stress is removed, the ink can be restored to a higher viscosity to avoid diffusion, so that the printed pattern is kept smooth and the traces on the screen printing are reduced. Suitable viscosity range for screen printing of ink is 500–5000 cp.^[^ ^81^ ^]^ The resolution and quality of screen printing largely depend on the stenciling techniques, the printability of the ink, and the affinity of the ink with the substrate. Furthermore, the number of frames, fabric mesh count, and plate thickness all play an essential role in controlling the final pattern resolution and the thickness of the printed film. Currently, the most advanced screen printing can reach a resolution of 30–50 µm,^[^ ^9^, ^66^ ^]^ which is sufficient for low‐demand electronic applications. In addition, the screen can be surrounded to form a tube around the ink supply and scraper. The synthetic tube roller can rotate and print at the same speed as the roll‐to‐roll machine, making screen printing continuous and high‐throughput manufacturing.

The main challenge of screen printing is to develop functional inks with appropriate rheological properties to facilitate the printing progress. The physical behavior of MXenes under stress defines the rheological property of MXene ink. Gogotsi and co‐workers^[^ ^82^ ^]^ conducted shear experiments on monolayer (ML) MXene sheet suspension and multilayer MXene dispersion with different concentrations to study the rheological properties of MXenes. The result shows that both suspensions showed apparent shear thinning behavior, and the viscosity increased with concentration. Single‐layer and multilayer MXenes have distinct rheological properties, and dispersions with different concentrations have extensive rheological properties, providing different printing possibilities for MXene ink. In addition, the size and concentration of 2D MXene material have a great influence on its rheological properties. The larger the size, the easier to adjust the rheological properties.

**Figure 3:**
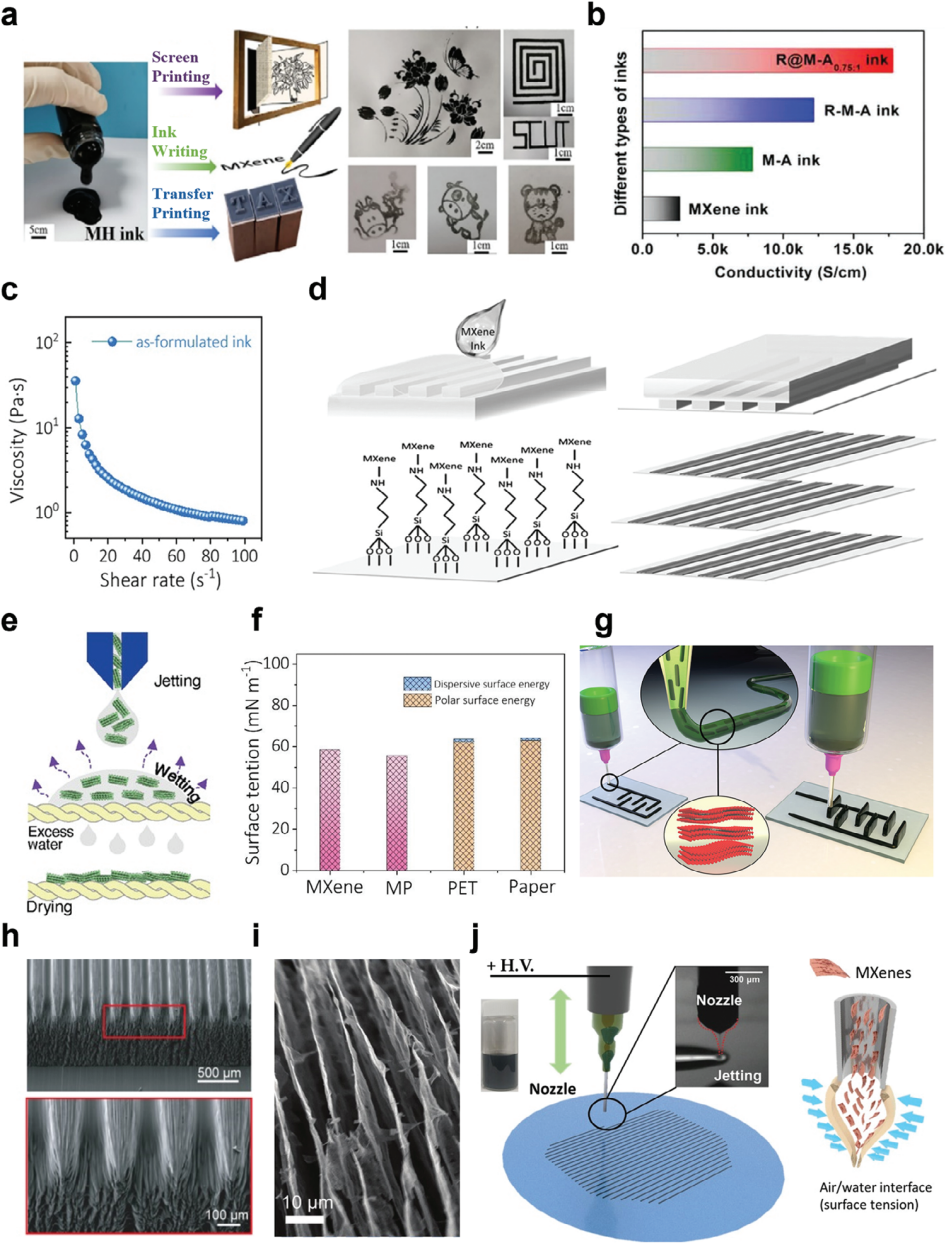
a) The image on the left shows MH ink and its printing strategy: screen printing, ink writing, and transfer printing. The right image is an image of a screen printing and transfer printing on cellulose paper. Reproduced with permission.^[^ ^9b^ ^]^ Copyright 2021, American Chemical Society. b) Electrode conductivity of RuO_2_·*x*H_2_O@MXene ink, M–A ink, R–M–A ink, MXene ink through screen printing. Reproduced with permission.^[^ ^9c^ ^]^ Copyright 2019, Wiley‐VCH. c) The figure on the left is the viscosity plotted as a function of shear rates of MXene inks. Reproduced with permission.^[^ ^66^ ^]^ Cpyright 2020, Wiley‐VCH. d) Illustration of the patterning process on glass substrates by using µCP. Reproduced with permission.^[^ ^83^ ^]^ Copyright 2020, Wiley‐VCH. e) Schematic of droplet formation during injecting followed by wet and drying of that substrate. Reproduced with permission.^[^ ^62a^ ^]^ Copyright 2020, Wiley‐VCH. f) Surface tension of M–K ink, MXenes, PET, fiber paper substrate. Reproduced with permission.^[^ ^21a^ ^]^ Copyright 2021, Wiley‐VCH. g) Schematic of MSC printing with interdigital architecture. The shear stress in the nozzle aligns the sheets horizontally in the direction of nozzle movement. Reproduced with permission.^[^ ^77^ ^]^ Copyright 2020, American Chemical Society. h) The figure shows the surface patterning by µCLIP combined with the surface topography of the microfeatures, and the DIW effect of the MXene/ethanol ink for orientation MXene assembly with anisotropic deposition and preferential alignment. Reproduced with permission.^[^ ^9a^ ^]^ Copyright 2021, American Chemical Society. i) The figure is a high‐magnification SEM image showing a high‐magnification SEM image of the layers with porosity aligned in the freezing direction (bottom‐up). Reproduced with permission.^[^ ^78^ ^]^ Copyright 2021, Wiley‐VCH. j) The schematic shows printing TFA–MX ink from nozzle electrohydrodynamic (ED). Reproduced with permission.^[^ ^10b^ ^]^ Copyright 2021, Wiley‐VCH.

The rheological properties, mechanical stability and electrical properties of MXene inks can be tuned by introducing binary functional additives. Qi and co‐workers^[^ ^9b^ ^]^ introduced a HCNF with typical shear thinning behavior to prepare MXene/HCNF (MH) ink, which is suitable for screen printing, direct ink writing, and transfer printing (**Figure** **3**a). The second component of HCNF successfully facilitates the extrusion of the MH ink and maintains the structure of the printed product. Moreover, HCNF with unique “core–shell” structure can promote the compatibility between HCNF and MXenes, which is conducive to the dispersion and stability of MXene conductive ink. Printable MXene ink can also serve as the “host” material for preparing of characteristic electrical devices by introducing other functional “guest” materials. Li et al.^[^ ^9c^ ^]^ introduced RuO_2_ nanoparticles on MXenes by an in situ synthesis strategy, and the resulting RuO_2_·*x*H_2_O@MXene sheet can associate with silver nanowires (AgNWs) to serve as a printable electrode with micrometer‐scale resolution for high performing, fully printed micro‐supercapacitors (MSCs). Both of the pure MXene ink, MXene/AgNW ink (M–A ink), and a RuO_2_·*x*H_2_O@MXene/AgNW (R–M–A) ink showed thixotropic properties, and screen‐printed interdigital electrodes by R–M–A ink showed higher conductivity (Figure 3b). In this printed nanocomposite electrode, RuO_2_ nanoparticles contribute high pseudocapacitance while preventing MXene nanosheets from restacking, resulting in the high volumetric capacitances of the obtained MSCs. In another work, an environment‐friendly strategy of turning MXene trash into treasure is adopted, the MXene sediments after minimally intensive layer delamination are collected to formulate screen printing ink without any additives (Figure 3c). The MXene trash ink exhibits an apparent viscosity of 35 Pa s, and a typical elastic‐like solid behavior. High‐efficiency printing of various patterns, such as conductive tracks and integrated circuits was realized through the above trash ink.^[^ ^66^ ^]^

##### Transfer Printing

Transfer printing is a strategy that transfers target materials from relief features on the intermediate medium to the target substrate, through which heterogeneous integration of different types of materials into the required layout was easy to implement, and has found huge applications in flexible/stretchable electronic products. Among them, direct transfer is similar to gravure printing, where the concave plate was coated with ink. In the transfer process, a scraper was used to scrape off the excess ink, leave the ink in the concave pattern part, and then apply pressure with rubber to make the ink stand out and transfer it to the printing materials. Direct transfer printing is constantly used in combination with a roll‐to‐roll process. Indirect transfer processes include sublimation, migration, melting, and ink layer stripping. Transfer of the film most utilizes a soft elastomeric stamp to modulate the physical mass transfer of the microdevice between the donor substrate and the recipient substrate.^[^ ^19^ ^]^ This microprocessing technique, which uses elastic seals combined with self‐assembling monolayer technology to print graphics on a substrate, is known as microcontact printing (µCP). µCP is suitable for various different surfaces, with no need for absolutely flat surfaces, and the printing accuracy reaches sub‐micrometer scale. The thickness of the micropattern is usually adjusted by controlling the concentration of the MXene ink^[^ ^83^ ^]^ (Figure 3d). However, MXene film is relatively fragile and easy to fracture in tensile deformations, which makes it unable to withstand the transfer process. Crack growth and slip between adjacent nanosheets can be inhibited by adding polymers and other nanomaterials.^[^ ^71^ ^]^ For example, single‐walled carbon nanotube (SWCNT), sodium dodecyl sulfate, and MXenes were mixed to prepare a planar MXene–SWCNT film by vacuum filtration, which is subsequently separated from the filter and transferred to the surface of an inflated latex balloon. Especially, the resultant MXene film and latex bonded together by interlocking structure, and stretchable electromagnetic interference (EMI) shielding and wearable antennas with complex pleated structures are successfully constructed using balloon‐constricted surface instability.^[^ ^84^ ^]^ The advantages of intaglio and flexographic transfer printing are fast printing speed and strong scalability. The main drawbacks are the high cost of setting up and prototyping the gravure printing roller and printing plate, as well as low printing accuracy (micrometer level). The improved adhesion strength of the film is also needed to promote long‐term stability, high compatibility requirements for ink materials, and the complex geometry of the substrate is not compatible.

#### 2.3.2 Noncontact Printing

##### Inkjet Printing

Inkjet printing is a noncontact, nonpressure, nonplate, high‐resolution digital printing technique, which has been widely used to fabricate various devices such as transistors,^[^ ^85^ ^]^ sensors, energy storage devices,^[^ ^21a^ ^]^ electromagnetic interference shielding,^[^ ^74^ ^]^ photodetector,^[^ ^64^ ^]^ organic light‐emitting diodes (OLEDs) and displays.^[^ ^86^ ^]^ Based on the droplet generation mechanism, inkjet printing is mainly divided into continuous inkjet printing and drop‐on‐demand inkjet printing (DOD‐IJP). DOD‐IJP based on the piezoelectric and thermal mechanism is the most commonly used inkjet printing method. The difference between the piezoelectric and thermal mechanisms is that piezoelectric inkjet printers utilize an electric field to generate pressure that pushes ink from the nozzle into individual droplets directed toward the substrate, while thermal printer operates by heating resistive element to form bubbles that can eject ink onto a substrate. In a typical piezoelectric printing process, the ink pump ejects the ink from the nozzle with a certain pressure to form a continuous ink flow, and then a large number of microink droplets are obtained by adjusting the pressure and voltage amplitude of the ink pump; the ink drop flow is charged through the high‐voltage electric channel, and the relatively large liquid drops are not charged. When the charging signal is removed, the charged microink drops are deflected under the action of the direct current field of the deflection electrode to form a printed ink beam, which is further sprayed on a printing substrate to complete the printing process. Meanwhile, noncharged ink droplets are not deflected and will be recycled by the ink system. Due to its special noncontact printing characteristics, inkjet printing can be compatible with various substrates, such as rigid silicon substrates and flexible polymer substrates, including cellulose paper, glass, polyethylene terephthalate (PET), and polymethylmethacrylate. Additive‐free aqueous MXene ink can be used for inkjet printing on various substrates and the evaporation rate can be accelerated by adding volatile solvent or using porous substrates such as textiles (Figure 3e). When hydrophilic cotton‐based textiles are used, the evaporation rate can be accelerated without needing additional pre‐ or postprocessing, which is essential for the facile construction of integrated circuits under ambient conditions.^[^ ^62a^ ^]^

There are several key issues that need to be addressed for fabricating functional MXene inks suitable for inkjet printing. i) Control the formation of stable droplets—inkjet printing requires careful tailoring of the viscosity and surface tension of the ink formulation. The unbefitting viscosity of the MXene ink will cause the formation of satellite droplets and jetting deflection, thus hindering the stability of the jetting process. Typically, inkjet printing requires low viscosity (1–10 cp) and appropriate surface tension over a narrow range (1–20 mPa s) to ensure stable jetting of single droplets.^[^ ^17^, ^87^ ^]^ ii) Large particles or agglomerates should be averted in the printing inks to avoid nozzle clogging. Typically, the particles in ink should be less than 1/50 of the diameter of the nozzle. In high resolution (10–20 µm) printing, small‐diameter printheads require lower viscosity of the ink. iii) Control the wetting and drying properties of the ink and the adhesion between the MXenes and substrate—in some cases, the ink and the substrate are not pinned down by the interaction force and printed patterns cannot be stable existed on the substrate. If the drying thermodynamics cannot be accurately controlled, it will flatten and form a “coffee ring,” which will lead to the nonuniformity of the print pattern, destroy the long‐term order in the sub‐micrometer structure, and reduce the quality of the print product.^[^ ^88^ ^]^ The application of solvents with low boiling points such as isopropyl alcohol in IJP ink can significantly reduce the coffee ring effect.^[^ ^23b^ ^]^ The interaction between MXenes and the substrate can be promoted by using binder molecule,^[^ ^89^ ^]^ proteins,^[^ ^74^, ^90^ ^]^ or a method for post‐treating the substrate, while improving the uniformity of the printed film. Single or mixed low boiling point alcohols (e.g., ethanol, 2‐butanol, isopropanol) and biologically compatible water are also proposed as the ink solutions for the modulation of ink surface tension and viscosity engineering.^[^ ^23^, ^85^, ^91^ ^]^ At the same time, the wetting and drying properties of the ink can be turned to obtain the appropriate morphology of the printed features. An aqueous printable MXene/poly(3,4‐ethylenedioxythiophene):poly(styrenesulfonic acid) (MP) hybrid ink can provide high conductivity, adjustable viscosity, resulting in remarkable printability and long‐term stability (Figure 3f). The addition of glycol to high concentration of aqueous MXenes reduced the viscosity and surface tension of the hybrid ink, and PH1000 (MP) in printed microelectrodes can effectively reduce the restacking of MXene layers and form conductive tunnel, thus promoting charge transfer, and ultimately contributing to the fabrication of microelectrodes with electrochemical and mechanical stability.^[^ ^21a^ ^]^

##### 3D Printing

3D printing is a manufacturing technique for rapid prototyping and customization of components and objects. Common 3D printing techniques include selective laser melting, selective laser sintering, fused deposition modeling, stereolithography, laminated object manufacturing, and direct ink writing (DIW).^[^ ^92^ ^]^ Among them, DIW based on printable inks technique can produce complex 3D structures at both mesoscale and microscale, and is the most effective building method for current electronic devices. DIW is suitable for depositing almost any material as long as the precursor ink has the appropriate rheological behavior.^[^ ^93^ ^]^ Conventional electrode manufacturing processes include dry coating, electrolyte filling, electrode scaling, electrode stamping, and heat sealing.^[^ ^94^ ^]^ DIW can avoid these processes, and achieves layer‐by‐layer printing and shape customization. In addition to minimizing material waste, DIW has more space‐efficient integration process. The commonly used DIW technology mainly includes pen‐based direct ink writing, extrusion printing, microcontinuous liquid interface production (µCLIP), and electrohydrodynamic (EHD) printing.

Pen‐based direct ink writing methods deposit ink using daily writing tools such as fountain pens and rollerball pens, and the printing resolution is in a range of 50–800 µm,^[^ ^95^ ^]^ suitable for simple operation of electronic fabrication and testing on lab‐scale. By contrast, extrusion printing allows digital and customized patterning, filaments are formed during extrusion through a nozzle and are deposited on a substrate layer by layer to form 3D complex geologic structure^[^ ^77^ ^]^ (Figure 3g). The viscosity of the viscoelastic ink is suitable for extrusion printing range from 10^6^–10^8^ cp and the printing resolution reaches almost 1 µm.^[^ ^96^ ^]^ Additive‐free MXene ink with high concentration has the characteristics of high viscosity and shear thinning, which is suitable for constructing multidimensional structures and devices through extrusion 3D printing. High‐performance electronic devices like micro‐supercapacitors^[^ ^97^ ^]^ and battery^[^ ^98^ ^]^ with thick electrodes, and self‐powered integrated sensing system^[^ ^18^ ^]^ and printed flexible wireless integrated sensing system^[^ ^2c^ ^]^ have been successfully constructed by customized printing MXene ink.

In order to realize the construction of hierarchical structures from nanoscale alignment to microscale patterning and then to complex macroscale landscape, more scalable hybrid printing methods need to be excavated to solve the problem of directional arrangement and patterning of MXene structures. For example, the µCLIP is used to construct the printing substrate. The oxygen permeable window between the photosensitive resins and the printing platform is used to form the oriented microstructure. Subsequently, the ink dropped during the DIW process is preferentially aligned along a specific printing path and controllable stacking density/packing factor to form an anisotropic and ordered micropatterning^[^ ^9a^ ^]^ (Figure 3h). For more miniature 3D structures, it is imperative to realize overhanging features or controllable cross‐sectional geometry by tedious chemical/thermal postprocessing. The fabrication of 3D aerogel structures using the 3D freeze printing (3DFP) method can not only compensate for the limitations of 3D printing, but also control the orientation of the nanosheets in both horizontal and vertical directions^[^ ^78^ ^]^ (Figure 3i). In particular, the unidirectional freeze casting process freezes a prepared gel or suspension of Ti_3_C_2_T*_x_* flakes by applying a unidirectional temperature gradient. Thus, ice crystals nucleate on cold surfaces and grow along the temperature gradients.^[^ ^99^ ^]^ Unlike extrusion‐based 3D printing, the entire process of 3DFP uses ice as the support material to construct 3D structures, and it does not require viscoelastic shear thinning inks, benefiting the formation of well‐ordered microstructures. At the end, the porous supercapacitors based on Ti_3_C_2_T*_x_* exhibit efficient ion transport, allowing for rapid electrochemical charge–discharge cycling.^[^ ^100^ ^]^

Noteworthy, EHD printing is a distinctive type of direct‐write printing, which can be used for the construction of high resolution, large area, flexible electronics components. The surface tension of ink plays a great role in maintaining the continuity of printing lines in this printing process. Considering the high surface tension of aqueous ink, the droplets must be pulled to the substrate by electrostatic force and high net charge capillary breaking force. Specifically, the droplets at the tip of the capillary form a Taylor cone under an applied external electric field and then elongate to form a jet stream.^[^ ^10^ ^]^ In addition, a continuous printing structure can be obtained by selecting an appropriate electrical voltage and nozzle‐to‐substrate distance. EHD printing inherits the advantages of inkjet, DIW, and electrospinning processes, such as low printing waste, the ability to print straight or serpentine nanofibers, printing efficiencies up to 1 m s^−1^, and the capability to deposit fibers directly onto silicon or polymer substrates.^[^ ^101^ ^]^ Moreover, EHD printing can only realize sub‐micrometer structure printing with sizeable inner diameter nozzles, which is two orders of magnitude higher than the resolution of inkjet printing. However, due to the limitation of printing materials, process technology, and environmental conditions, the quality of EHD printing products is closely related to the molecular weight, concentration, viscosity, conductivity, dielectric constant, surface tension, temperature, and humidity of the printing environment.^[^ ^101^ ^]^ MXenes treated with trifluoroacetic acid (TFA–MX) can be used for EHD printing by employing ethanol as solvent. TFA–MX inks facilitated the manufacturing on flexible or rigid substrates without any pretreatment,^[^ ^10^, ^17^ ^]^ and delicate patterns with high resolution were produced by EHD printing (Figure 3j). The stable dispersion of MXenes in organic solvents remains excellent electrical properties, and are highly compatible with EHD printing.

Therefore, for MXenes, printing technique not only means a set of general, scalable, reproducible, and cost‐effective pattern methods, but also is a key factor that researchers of MXenes need to pay attention to in the cycle of ink synthesis, modification, and application. The printing resolution, thickness, and roughness of the resultant patterned film/architecture highly depend on many factors,^[^ ^102^ ^]^ including printing process (inkjet printing, spraying, gravure printing, 3D printing, etc.), physical properties of printable ink (i.e., rheology, viscosity, inherent electrical properties, wettability, etc.), surface properties of substate (morphology, wettability, reactivity, etc.), and post‐treatment conditions.^[^ ^103^ ^]^ Besides these printing‐related issues, MXene contacts (surface microstructures, contact defects, and energy level matching) existing in multiple layered printed electronics are also critical factors impacting the device performance,^[^ ^104^ ^]^ which will be discussed in more detail in subsequent chapters. By carefully modulating the contact engineering between functional layers, the performance and application of MXene‐based devices will be dramatically expanded. When these two features are combined, the high‐throughput and green manufacturing of scalable, wearable, and low‐cost integrated electronic products with high performance is possible.

## 3 Contact of MXene Electrodes with Various Semiconductors

**Figure 4:**
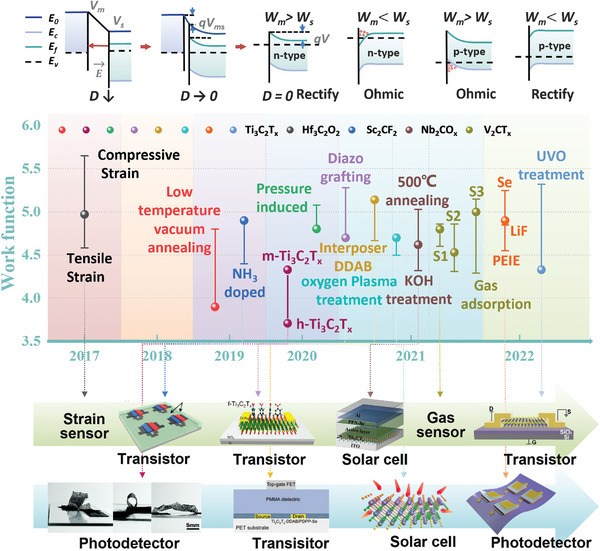
Metal–semiconductor contact energy band diagram. They have independent vacuum energy level (*E*_0_), the conduction band (*E*_c_), the valence band (*E*_v_), and the Fermi energy level (*E*_f_) before contact. “*D*” denotes the distance between them. As the distance decreases, the energy band is bent. According to the difference of carrier type and the contact barrier height, it is divided into n‐type contact and p‐type, rectifying contact and Ohmic contact. MXene work function regulation approaches, range and the corresponding application scenarios in the past five years, including compressive strain, tensile strain,^[^ ^105^ ^]^ low temperature vacuum annealing,^[^ ^106^ ^]^ ammonia solution mixed doping,^[^ ^107^ ^]^ pressure‐induced,^[^ ^108^ ^]^ diazo grafting pulling impregnation,^[^ ^2b^ ^]^ interposer DDAB embedded adsorption,^[^ ^109^ ^]^ oxygen plasma treatment,^[^ ^110^ ^]^ annealing at 500 °C, KOH treatment,^[^ ^111^ ^]^ thermal evaporation Se and LiF, mixed with PEIE,^[^ ^104^ ^]^ ultraviolet ozone (UVO) treatment.^[^ ^112^ ^]^ H‐MXene and m‐MXene stand for the MXene synthesized through different post‐treatment processes, including HF etching and TBAOH intercalation method (h‐MXene) and the minimally intensive layer delamination (MILD) method (m‐MXene).^[^ ^113^ ^]^ Gas adsorption (S1, S2, S3) stand for different configurations reflecting different synthesis conditions: V_2_C(OH)_0.22_F_0.44_O_0.33_, V_2_C(OH)_0.33_F_0.11_O_0.55_, and V_2_C(OH)_0.33_F_0.05_O_0.61_ plus H_2_O molecule.^[^ ^114^ ^]^ The sphere and error bar, respectively, represent the initial work function value and the regulated work function value or range. Top line from left to right: Reproduced with permission.^[^ ^107^ ^]^ Copyright 2019, American Chemical Society. Reproduced with permission.^[^ ^2b^ ^]^ Copyright 2021, American Chemical Society. Reproduced with permission.^[^ ^111^ ^]^ Copyright 2021, American Chemical Society. Reproduced with permission.^[^ ^112^ ^]^ Copyright 2022, Wiley‐VCH. Bottom line form left to right: Reproduced with permission.^[^ ^113^ ^]^ Copyright 2020, Royal Society of Chemistry. Reproduced with permission.^[^ ^109^ ^]^ Copyright 2021, Wiley‐VCH. Reproduced with permission.^[^ ^110^ ^]^ Copyright 2021, American Chemical Society. Reproduced with permission.^[^ ^104^ ^]^ Copyright 2022, Wiley‐VCH.

The MXene family has been considered as a class of evolving 2D nanomaterials, attributed to the superiorities of the outstanding conductivity, abundant terminal groups, hydrophilicity, large surface, and unique layered structure, as discussed in above sections. In the recent five years, many pioneering works have committed to the development of MXene‐based composites with various functions and designed electronic structures, and diverse device application (**Figure** **4**). On the one side, the metallic, semiconducting properties, and topological properties of MXenes can be determined by modulating the surface groups to change the energy band structure, and its electrical and mechanical properties can also be intervened employing intercalation and chemical modification. On the other hand, for many electronic devices, the type of contact between functional materials highly affects the performance and conversion efficiency of the device. Thus, the surface properties of MXenes and the contact properties with other materials are critical factors in implementing the applications of printed electronics.

### 3.1 Electronic Properties of MXenes

#### 3.1.1 Calculation and Simulation

The generalized gradient approximation (GGA), density functional theory (DFT), density‐functional‐theory‐based tight binding, molecular dynamics (MD) simulation, and other computational methods are commonly combined to conduct systematic research on electronic properties of MXene materials. As reported by Di Carlo and co‐workers,^[^ ^115^ ^]^ based on DFT calculations, the mixed terminations of —F, —OH, and/or —O affect the work function of Ti_3_C_2_ MXenes, covering the whole phase space of mixtures. In addition, to address the relatively simple computational problems, the hybrid functionals proposed for solid‐state calculations by Heyd–Scuseria–Ernzerhof (HSE06) can be used to accurately estimate the bandgap.^[^ ^116^ ^]^ For example, Chuang and co‐workers^[^ ^117^ ^]^ used HSE hybrid functional calculations to show fluorinated MXenes with sizable nontrivial bandgaps from 34 to 318 meV. The popular DFT dispersive correction (DFT‐D) semiempirical dispersion correction method was adopted to deal with vdW effects.^[^ ^118^ ^]^ Based on this method, —OCH_3_‐functionalized Nb*_n_*_+1_C*_n_* MXene sheets were suitable for the field emitters with an ultralow work function of 1.0 eV.^[^ ^4a^ ^]^

Quantitative evaluation of the bandgap and optical spectrum of the MXenes can be achieved by solving the quasiparticle GW (G and W are the dynamically screened Coulomb interaction and the dressed Green’s function, respectively) approximation of the Bethe‐Salpetere (BSE) equation.^[^ ^119^ ^]^ Considering the electronic interaction in the transition metal, the anti‐ferromagnetism related to orbital ordering property can be obtained by the use of generalized DAT Hubbard model (DFT+*U*) functional independent of base set,^[^ ^120^ ^]^ and the structural relaxation and electronic structure calculation can be carried out under the consideration of the strong correlation effect in the transition metal.^[^ ^116^, ^121^ ^]^ To include the exciton effect, the optical properties are discussed using the time‐dependent Hartree–Fock calculations.^[^ ^122^ ^]^ Unless otherwise specified, the following discussion of the properties of MXenes employs one or more of the above computational models in combination with analysis.

#### 3.1.2 Conduction Mechanism of Intrinsic MXene

**Figure 5:**
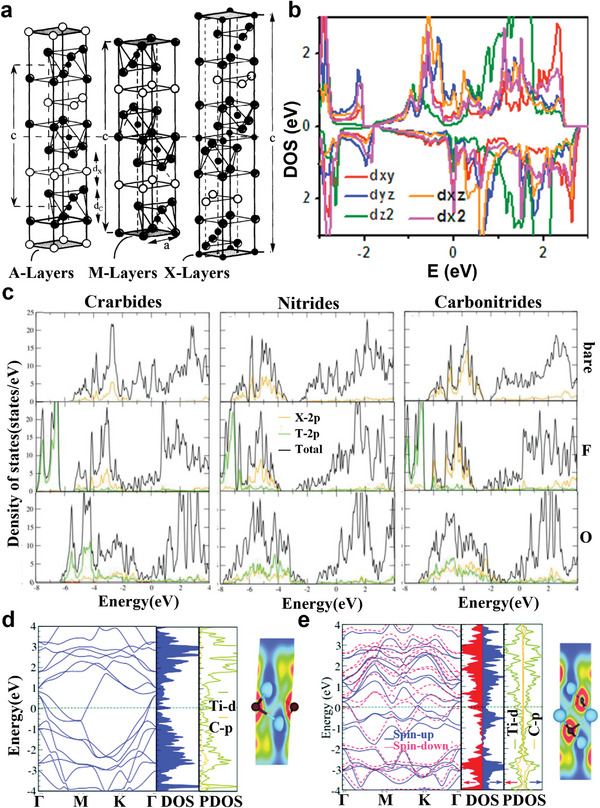
a) Unit cells of 211, 312, and 413 phases in sequence from left to right. The unit cell is depicted by vertical arrow labeled c and the horizontal dashed line is drawn through the center of the unit cell. Reproduced with permission.^[^ ^123^ ^]^ Copyright 2000, Elsevier. b) Plotted partial DOS of d‐orbitals for Ti atoms in pristine Ti_3_C_2_ monolayer. Reproduced with permission.^[^ ^124^ ^]^ Copyright 2015, Elsevier. c) The projected density of states (PDOS) and total density of states (DOS) for Ti_3_C_2_, Ti_3_N_2_, and Ti_3_CN of the bare (first row), —F‐terminated (second row), and —O‐terminated (third row) MXenes. The total DOS, C/N‐2p (X‐site), and T‐2p (terminal) states are shown in black, orange, and green curves, respectively. The middle and surface titanium atoms are represented by red and blue curves. For bare MXenes, the DOS color of the additional type of Ti atom is cyan. The Fermi level is set to 0 eV. Reproduced with permission.^[^ ^7^ ^]^ Copyright 2020, Wiley‐VCH. Electronic structure and PDOS of d) Ti_2_C and e) Ti_3_C_2_ monolayers. Contour plots of the electron localization function (ELF). The red (blue) region denotes high (low) electron density. Reproduced with permission.^[^ ^125^ ^]^ Copyright 2021, Royal Scoiety of Chemistry.

The MAX phase has a layered hexagonal structure, the space group is $D_{6h}^{4}$–*P*6_3_/*mmc*, and the chemical formula is M*_n_*_+1_AX*_n_* (*n* = 1–3), where “A” represents an element from groups 13–16 (Al, Si, P, S, Ga, Ge, As, In, and Sn) of the periodic table. In each MAX unit cell, the closely packed M layers are interleaved with the A layers, and X atoms fill the octahedral sites of the M layers. **Figure** **5**a compares the unit cell structures of 221, 312, and 413. The main difference is in the number of M layers separating the A layers, there are two in the 211 phase, three in the 312 phase, and four in the 413 phase.^[^ ^123^ ^]^ Similar to the precursor of MAX, the energy level of the bare MXenes near the Fermi surface is dominated to a large extent by the d‐electrons of the transition metal atom, and the p‐electrons of the X atom form an energy band near −3 to −5 eV below the Fermi level^[^ ^124^ ^]^ (Figure 5b). The outer transition metal layer of MXenes plays a more essential role in the electronic properties than the inner core transition metal layer. The total and orbital projected density of states (DOS) of the carbide and nitride MXenes are shown in Figure 5c. The existence of additional 2p electrons in nitrogen atoms makes the density of states of Fermi energy levels of nitrides higher than that of carbides. From the electronic structures of carbides and nitrides in MXenes, the surface charge of Ti_3_N_2_ is less than that of Ti_3_C_2_ by about 0.1*e* without termination, and the Bader charge of Ti atom in the interlayer is less sensitive to surface groups. According to the energy band diagram and projected density of states (PDOS) of different MAX phases, a high state density was observed near the Fermi level showing good metal performance.^[^ ^7b^ ^]^ Combined with the computational results, Ti_2_C and Ti_3_C_2_ show the behavior of metal and ferromagnetic metal in the ground state, respectively. The contribution of transition metal is still greater than C atom at the Fermi level (Figure 5d). Correspondingly, the electron density around Ti atom and C atom presents the enrichment state and depletion state^[^ ^125^ ^]^ (Figure 5e).

The results of electronic structure and molecular dynamics calculations based on density functional theory as well as neutron scattering indicate that the greater electronegativity of the N atom causes the stronger Ti—X covalent bond. Therefore, the lattice parameters of nitrides, in both bare MXenes and surface‐terminated MXenes, are smaller than those of carbides. Compared with the pristine MXenes, by expanding the electronic state and varying the electrostatic potential distributions, the carrier transport capability of the surface functionalized sample is significantly enhanced.^[^ ^126^ ^]^ Near the Fermi level, the electron transport capability of the fluorinated system is stronger than that of the hydrogenated system. At the position far away from the Fermi level, the transport levels of the two systems are equivalent, the extension of the electronic state between the electrodes can be observed, which dramatically increases the possibility of electron transfer through the system.^[^ ^7^, ^124^ ^]^ While the bare and oxidized electronic states remained mostly near the MXene electrodes, the passing through of electrons will be reduced, resulting in lower probability of electron transport.

#### 3.1.3 Regulation of MXene Bandgap

Under the natural etching conditions of MXenes, —F, —O, —OH, and other functional groups are easily adhered to the surface of the M atoms. Surface functionalization can contribute to the regulation of the bandgap of MXenes, and even turn the metallic MXenes into semiconductor. The calculated band structures indicate that the semiconductors Ti_2_CO_2_, Zr_2_CO_2_, Hf_2_CO_2_, and Sc_2_CF_2_ have an indirect bandgap, and Sc_2_C(OH)_2_ has a direct bandgap, while the band structure of its corresponding 2D form depends on the position of the functional group.^[^ ^127^ ^]^ In addition, the band structure of MXenes can be modified by applying external strain,^[^ ^128^ ^]^ electric fields,^[^ ^118b^ ^]^ gate voltages,^[^ ^129^ ^]^ atomic doping,^[^ ^130^ ^]^ and so on.

**Figure 6:**
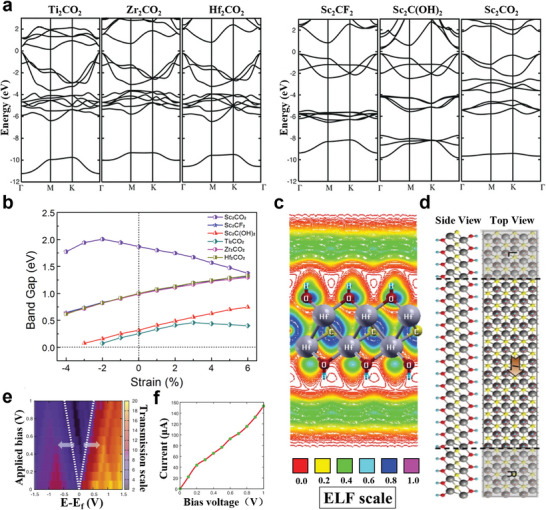
a) Band structure of Ti_2_CO_2_, Zr_2_CO_2_, Hf_2_CO_2_, Sc_2_CF_2_, Sc_2_C(OH)_2_, and Sc_2_CO_2_ systems. The Fermi energy is at zero. Reproduced with permission.^[^ ^124b^ ^]^ Copyright 2012, Wiley‐VCH. b) Relationship between bandgap of MXene‐functionalized monolayers and applied biaxial strain. Positive and negative abscissa values correspond to tensile and compressive strain, respectively. Reproduced with permission.^[^ ^116c^ ^]^ Copyright 2016, IOP Publishing. c) ELF contour plot for Hf_2_C(OH)_2_. d) Schematic representation for the electronic transport calculation of Hf_2_C(OH)_2_. The regions by L and R symbols represent the semi‐infinite left and right electrodes. e) Transmission spectrum of Hf_2_C(OH)_2_ at different bias voltages. f) Current–voltage characteristics of Hf_2_C(OH)_2_. The Fermi level (*E*_f_) is at the zero level. Reproduced with permission.^[^ ^138^ ^]^ Copyright 2016, Wiley‐VCH.

Researchers^[^ ^116^, ^124^ ^]^ have studied the most stable configurations of semiconductor MXenes, which are mainly divided into two categories, the configuration (I) Ti_2_CO_2_, Zr_2_CO_2_, Hf_2_CO_2_, Sc_2_CF_2_, and Sc_2_C(OH)_2_ monolayers, and the configuration (II) Sc_2_CO_2_ monolayer (**Figure** **6**a). For above semiconductor MXenes, the energy gap fluctuates in the range of 0.24–1.8 eV.^[^ ^124b^ ^]^ Since Ti, Zr, and Hf belong to the same group in the periodic table of elements, and have the same number of shell electrons, the corresponding MXene systems show similar metal to semiconductor behavior near Fermi energy under the same functionalization type. The overall trend of the influence curve of biaxial strain on the bandgap value of semiconductor MXenes shows that with the increase of the compressive strain, the bandgap decreases continuously^[^ ^116c^ ^]^ (Figure 6b).

Carrier mobility, as a critical parameter of the semiconductor devices, can be obtained from the calculated electronic properties. According to the model, the mobility needs to be calculated based on the carrier effective mass. The relatively low electron effective mass is caused by the strong band dispersion in the conduction band relating to the weak variation of the atomic potential in the bare MXene plane.^[^ ^131^ ^]^ The semiconductor MXene material can refer to the mobility and anisotropy of electrons and holes to guide the doping type and application scenarios. For example, the hole mobility in Lu_2_C(OH)_2_ is much lower than the electron mobility, so n‐doping in Lu_2_C(OH)_2_ is more appropriate. By contrast, the electronic wave function of Lu_2_CF_2_ is more localized under small uniaxial strains, and the difference in energy level leads to higher deformation potential and lower carrier mobility.^[^ ^132^ ^]^ The calculated results show that Ti_2_CO_2_/Hf_2_CO_2_ has the highest carrier mobility in all MXenes, and the hole mobility of Ti_2_CO_2_ in the *x*‐ and *y*‐directions is two orders of magnitude higher than that of electrons. This high directional anisotropic carrier mobility can be used to separate electrons and holes in photocatalysis, reducing the recombination rate of electron hole pairs.^[^ ^133^ ^]^ The calculation results of Ti_2_CO_2_/Hf_2_CO_2_ vdW heterostructure show that on one side of the interface, the electrons show a dominant tendency along the *y*‐direction, and the holes tend entirely to migrate along the *x*‐direction on the other side.^[^ ^122^ ^]^ There was no significant difference in the deformation potential constants when compared to the monolayer ScCCl_2_, while the elastic modulus was increased by a factor of 2–3. The maximum carrier mobilities of the final electrons and holes in the *x*‐direction were 4.5 × 10^4^ and 1.0 × 10^4^ cm^2^ v^−1^ s^−1^, respectively.

Notably, some of these MXenes exhibit carrier mobility equivalent to or even high than well‐known 2D materials, such as Lu_2_C(OH)_2_ and Nb_2_CN_2_, high carrier mobility often implies high leakage current in transistor devices, which has potential application value in high‐speed electronic devices.^[^ ^131^ ^]^ The specific calculated parameter values for MXenes are shown in **Table** **2**. Based on these theoretical calculations, the electronic and carrier mobility of various MXenes and other optical, thermal, and magnetic properties can be predicted. It not only shows the effectiveness of surface properties in modulating the electronic properties, but also provides a reference for the application of the MXene family to electronic devices, making MXenes a desirable candidate for the design of nanoelectronic devices.

**Table 2:** Carrier types “e” and “h” denote “electron” and “hole,” respectively. For hole mobility, there are two quasi‐degenerate sub‐bands at valence band maximum (VBM), which are labeled as “h1” and “h2,” respectively. $m_{x}^{*}$ and $m_{y}^{*}$ are the effective masses along the *x* (zigzag) and *y* (armchair) directions. *E_x_* and *E_y_* are the deformation potential constants, and *C_x_* and *C_y_* are the elastic modulus. *µ_x_* and *µ_y_* are the room‐temperature carrier mobilities. The 1L, 2L, and 3L represent one layer, two layers, three layers, respectively

System	Carrier type	m*/*m*_0_		*E_x_* [ev]	*E_y_*	*C_x_* [J m^−2^]	*C*_*y*_ [J m^−2^]	*u_i_* [cm^2^ V^−1^ s^−1^]		Ref.
		$m_{x}^{*}/m_{0}$	$m_{y}^{*}/m_{0}$					*u_x_*	*u_y_*	
Lu_2_CF_2_	e	0.197	1.10	5.65	5.60	154	155	1120	206	[132]
	h1	3.06	3.75	4.68	−1.78	154	155	14.4	81.1	
	h2	0.332	0.348	5.00	−2.11	154	155	1.17	6.29	
Lu_2_C(OH)_2_	e	0.280	0.231	−0.696	−0.507	154	154	95.1	217.1	
	h1	2.55	2.69	−6.374	0.182	154	154	0.0121	14.01	
	h2	0.185	0.163	−6.374	−6.037	154	154	2051	2.91	
Sc_3_(CN)F_2_	e	0.21	1.70	5.722	4.144	262.15	–	1348	319	[134]
	h1	2.93	2.81	4.422	4.622	262.15	–	78	78	
	h2	0.51	0.53	−3.098	−2.894	262.15	–	956	1003	
Hf_2_CO_2_	e	0.231	2.162	10.57	7.101	293.6	291.0	329	77	[116d]
	h1	0.423	0.164	7.636	2.297	293.6	291.0	924	260	
	h2	0.164	0.414	2.023	7.442	293.6	291.0	343	100	
TiCO_2_/Zr_2_CO_2_	e	2.448	0.387	3.120	4.700	484	484	118.147	2083.232	[122]
	h	0.192	0.367	2.000	2.220	484	484	46 740.451	10 382.867	
TiCO_2_/Hf_2_CO_2_	e	2.483	0.437	2.780	4.340	528	533	157.798	1801.090	
	h	0.181	0.445	1.980	1.940	528	533	58 540.494	10 183.890	
Fe_2_CO_2_	e	6.99	2.45	1.63	1.04	211.41		53.23	378.80	[135]
	h	0.66	1.10	1.28	1.50	214.09		4455.42	1975.94	
Co_2_CF_2_	e	1.06	7.54	6.94	4.08	168.89		22.69	69.67	
	h	0.64	1.72	4.52	3.97	169.29		238.61	115.39	
Co_2_C(OH)_2_	e	2.01	0.59	2.30	2.22	185.99		184.06	240.17	
	h	0.59	0.39	2.73	1.19	189.86		3573.28	13 891.38	
Ni_2_CF_2_	e	0.34	3.85	1.77	3.08	137.76		2192.84	64.43	
	h	0.30	1.78	3.78	2.61	138.86		853.88	304.63	
Ti_2_CO_2_	e	0.44	0.53	5.71	0.85	267.27	265.21	611	254	[133]
	h	0.14	0.16	1.66	2.60	267.27	265.21	74 100	22 500	
Posphorene	e	1.12	0.124	5.34	2.79	103	24.5	177	1380	[136]
Sc_2_CF_2_	e	0.253	1.46	2.26	1.98	193	182	503	1070	
	h1	2.25	1.46	1.91	−4.17	193	182	483	386	
	h2	0.461	0.438	−5.00	2.20	193	182	309	261	
Sc_2_C(OH)_2_	e	0.496	0.494	−2.65	−2.57	173	172	206	219	
	h3	5.01	0.269	−3.46	−9.94	173	172	51	114	
	h4	0.290	1.91	−10.0	−2.23	173	172	164	238	
Nb_2_CN_2_	e	0.11	0.06	0.44	1.51	409.52	844.15	4 755 000	1 526 000	[137]
	h	0.11	0.05	5.06	4.87	–	–	25 000	95 000	
Ta_2_CN_2_	e	0.11	0.08	8.82	8.26	458.10	940.27	12 000	37 000	
	h	0.10	0.06	1.85	2.45	–	–	318 000	621 000	
ScCCl_2_ (1L)	e	0.23	1.319	1.17	1.19	158.9	161.1	19 277	3351	[131]
	h	0.685	2.260	1.88	1.79	158.9	161.1	1127	380	
2L	e	0.231	1.337	1.07	1.04	311.9	316.7	45 342	8421	
	h	0.412	1.809	2.05	2.03	311.9	316.7	4437	1047	
3L	e	0.232	1.325	1.40	1.34	475.8	479.8	40 457	7806	
	h	0.379	1.887	1.76	1.76	475.8	479.8	10 227	2074	

By using the first principle, it is found that nearly free electronic (NFE) states of some —OH‐terminated MXenes (Ti_2_C(OH)_2_, Zr_2_C(OH)_2_, Zr_2_N(OH)_2_, Hf_2_C(OH)_2_, Hf_2_N(OH)_2_, Nb_2_C(OH)_2_, and Ta_2_C(OH)_2_) exist near the Fermi level and show partial occupancy, while the NFE of graphene, graphite, MoS_2_ are above the Fermi level, indicating the unoccupied state (Figure 6c). The NFE near the vacuum level is not suitable for electron transport, and the occupied NFE band can facilitate electron–phonon coupling. Further research suggests that Hf_2_C(OH)_2_ can become an ideal transmission channel without nuclear scattering for electron transport.^[^ ^138^ ^]^ The positions of NFE states are outside the atomic structure of MXenes, and extend parallel to the surfaces (Figure 6d). With the further increase of the compressive or tensile strain, the partially occupied NFE state near the Fermi level eventually disappears. In addition, the transmission probability of the partially occupied NFE decreases when the bias voltage is applied, and disappears when the bias voltage > 0.6 V (Figure 6e,f). The external electric field can also regulate the energy of the NFE state, resulting in the transition from semiconductor to metal of Sc_2_C(OH)_2_.^[^ ^139^ ^]^ These methods based on the control of bandgap extend the application of MXenes to electronic switches.

The energy band structure of MXenes is susceptible to element composition due to the SOC effect since the transition metals (Mo, W, Zr, and Hf) in MXenes have relatively heavy 4d and 5d orbitals. The theoretical study indicates that MXenes can realize the metal‐to‐semiconductor transition, but also proposes that some MXenes with a wide bandgap will be topological insulators.^[^ ^140^ ^]^ The predicted nontrivial topological states of MXenes are revealed by the *Z*2 index, which is evaluated by the parity of the occupied band below Fermi level and the existence of the edge state at time‐reversal invariant momentum. At present, most of the studies on the electronic and structural applications of many MXene materials are based on theoretical calculations. Many experimental studies related to the predicted properties need to design comprehensive experiments to verify, while exploring more application scenarios such as ferromagnetic or topological insulators.

### 3.2 Surface Functionalization of MXene

Surface functionalization of MXenes by chemical modification or physical blending as mentioned in above sections allows for regulating its energy band structure, surface dipole, reducing defects, matching energy levels, and thus enhancing the performance of the electronic devices. In addition, for electrical energy storage, negatively charged functional groups will hinder electrolyte transport and cause problems such as oxidation,^[^ ^141^ ^]^ and the incompatibility with hydrophobic polymer and MXenes results in low interfacial bonding strength and poor mechanical properties, thus the surface property is particularly essential. Although mentioned in the previous section, we have listed a separate section to discuss the relevant role.

By manipulating the coordination of the MXene surface termination groups, the modulation of their energy storage capacity,^[^ ^142^ ^]^ magnetism,^[^ ^124^, ^143^ ^]^ bandgap,^[^ ^125^ ^]^ surface plasmon resonance,^[^ ^144^ ^]^ and carrier transport^[^ ^145^ ^]^ can be implemented, and thus a more reasonable structure can be designed for specific applications.

#### 3.2.1 Termination and Group Identification

**Figure 7:**
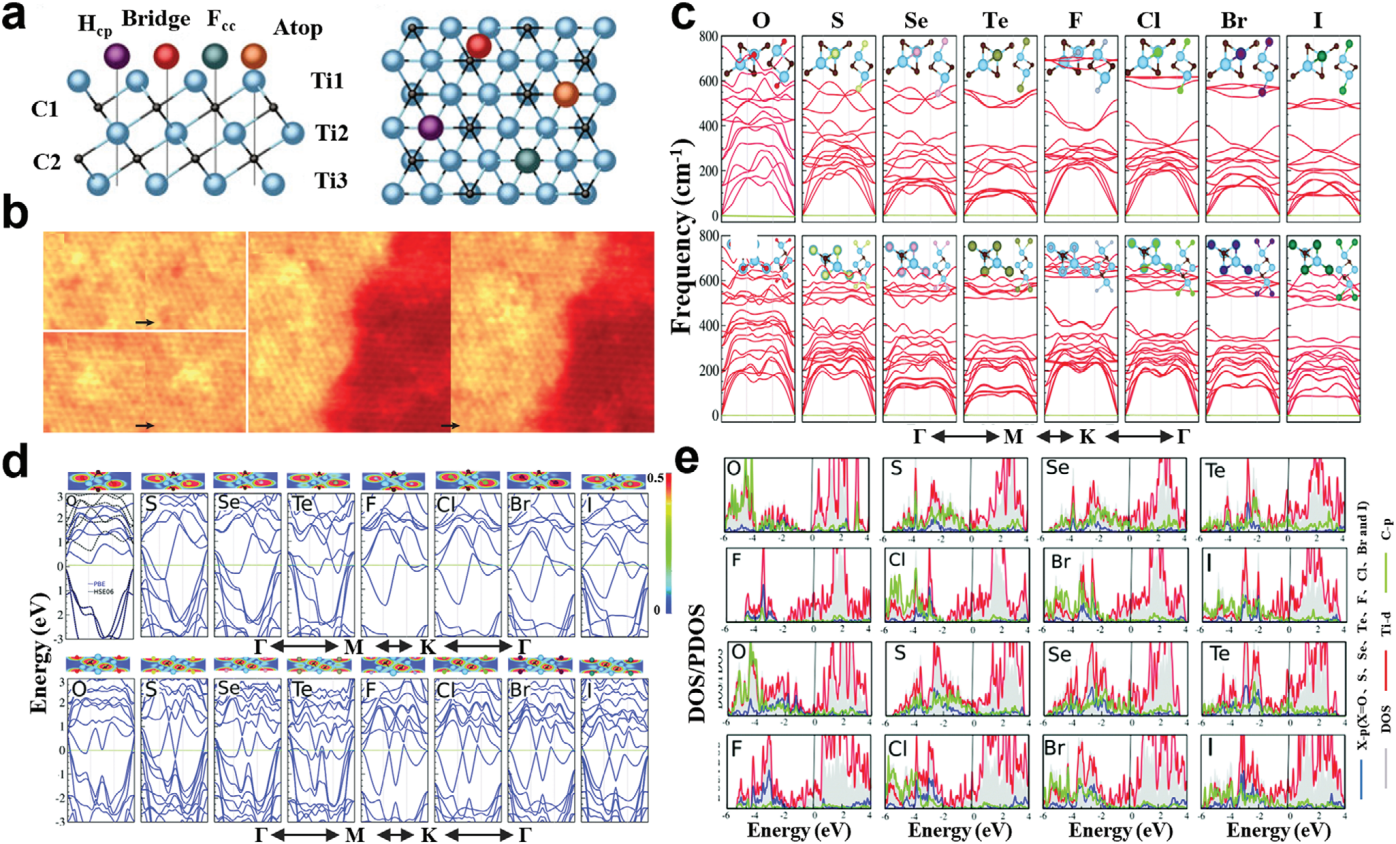
a) Available high‐symmetrical positions of the surface termination species on M_3_X_2_ as shown in side view (left) and top view (right): Atop, top of the titanium atom; Bridge, between two top titanium atoms; F_cc_, intermediate layer titanium atom above; and H_cp_, above that carbon atom. b) Local dynamics on Ti_3_C_2_T*_x_* thin plates and the mages were acquired 60 s apart. The left is a vacancy in a single sheet with the second sheet in projection. The middle is Adatom in a single sheet with a second sheet in projection. The right is at a step edge. Reproduced with permission.^[^ ^146^ ^]^ Copyright 2019, Springer Nature. c) Phonon spectrum of the functionalized Ti_2_C (top) and Ti_3_C_2_ (bottom) monolayers. Insets are top and side views of the atomic structures of the monolayers. d) DOS and PDOS of functionalized Ti_2_C (top) and Ti_3_C_2_ (bottom) monolayers. The zero of energy is set at the Fermi energy. e) Electronic band structure of functionalized Ti_2_C (top) and Ti_2_CO_2_ (bottom) monolayers. The corresponding DOS and PDOS are shown at the top of the electronic band structures. The zero of energy is set at the Fermi energy. Reproduced with permission.^[^ ^125^ ^]^ Copyright 2016, Royal Scoiety of Chemistry.

The surface functional groups of MXenes induce the formation of surface dipoles, giving rise to the movement of the vacuum energy level. Therefore, for pure MXenes, the energy band structure can be controlled by intervening the proportion of surface functional groups. Theoretically, there are different possibilities for the surface termination position of MXene sheets. For —O, —OH, and —F terminals,^[^ ^146^ ^]^ they were found to be more inclined to occupy the F_cc_ position, because of the steric repulsion between the terminals and the X element at the H_cp_ site (**Figure** **7**a).

The terminals of the bilayer Ti_3_C_2_T*_x_* MXenes are highly dynamic at ambient temperature.^[^ ^146^ ^]^ As shown in Figure 7b, the terminating species on the surface of MXenes moves in the form of clusters. During the slow oxidation process, MXene is gradually transformed into metal oxyfluorides at —F terminal sites, resulting in the decrease of —F terminal and increase in the number of —O terminals. At different temperatures, the migration of O signal can be seen as the oxygen signal reorganized with changes in surface temperature. Figure 7c is the phonon spectra of Ti_2_C and Ti_3_C_2_ after complete chemical modification with sulfur and halogen atoms. The frequency of the optical modes in the phonon frequency depends strongly on the radius of the halogen atom. Furthermore, there is no negative frequency in the phonon spectrum, indicating that all the configurations of the functionalized MXenes with the sulfur and halogen atoms are dynamically stable. In the calculated energy band diagram of functionalized MXenes by halogen atoms (X = F, Cl, Br, I) and chalcogen atoms (X = S, Se, Te) (Figure 7d), Ti_2_C is fully functionalized by the O atom, which could be attributed to the hybridization of d‐orbitals of carbon atoms and p‐orbitals of oxygen atoms. Thus, the energy gap decreased to 0.25 eV. Ti_2_C is functionalized by oxygen atoms into semiconductors and reduced to Perdue‐Burke‐Ernzerh (PBE) levels, while remaining metal after functionalization of other atoms. Among them, the p‐orbitals of the transition element dominate the formation of the conduction band, and the C‐p orbitals act on the valence band. The appearance of the bandgap is more due to the hybridization of the d‐orbitals of C atom and p‐orbitals of O atom. Not only that, the p‐orbital of the X atom also contributes the valance. Notably, the halogen atoms have a more negligible effect on the carbon orbital near the Fermi level than the chalcogen atoms^[^ ^125^ ^]^ (Figure 7e).

In the experiment, because the surface of MXenes is pretty complex and highly sensitive to the nature and structure of atoms, etching conditions, intercalation methods, and storage environment, it is challenging to accurately identify the types and numbers of surface terminals of MXenes. Currently, many characterization technologies have been adopted to achieve the aforementioned goal, including X‐ray photoelectron spectroscopy (XPS), nuclear magnetic resonance (NMR), X‐ray atom pair distribution function (X‐ray PDF), X‐Ray Diffraction (XRD), electronic energy loss spectrum (EELS), etc.^[^ ^147^ ^]^ Among them, XPS is widely used to determine the surface chemical compositions and chemical states of various species, thus enabling the identification and separation of —O and —OH.^[^ ^106^, ^110^, ^148^ ^]^ NMR,^[^ ^149^ ^]^ a complementary method for probing the chemical and electronic structures of surface binding terminals, can be used for the identification of the interactions of termination groups.^[^ ^150^ ^]^ X‐ray PDF can model the periodic and hybrid termination of bare MXene sheets and analyze the interlayer exchange and potential hydrogen bond interactions between the termination.^[^ ^151^ ^]^ EELS has been used for quantification, but for site identification and identification of functionalized species.^[^ ^141^, ^152^ ^]^ Scanning transmission electron microscopy (STEM) is used as a supplemental method for identifying and quantifying O and F.^[^ ^106^, ^153^ ^]^ Potential approaches also include neutron scattering,^[^ ^7^, ^154^ ^]^ Raman spectrum,^[^ ^155^ ^]^ surface acoustic detection,^[^ ^156^ ^]^ thermogravimetric analysis coupled with mass spectroscopy,^[^ ^157^ ^]^ and so on.

#### 3.2.2 Physical and Chemical Modulation of Terminals

##### Etching Method Selection

The type and proportion of surface functional groups of MXenes are highly dependent on different synthesis methods. The terminal proportion of the TiCT*_x_* products identified and quantified by H and 19F NMR experiments and spin counting is Ti_3_C_2_(OH)_0.06_F_0.25_O_0.84_ for LiF–HCl method produces, while Ti_3_C_2_(OH)_0.12_F_0.8_O_0.54_ for HF method. It was found that HF‐synthesized MXenes contained ≈4 times more F terminations compared to MXenes synthesized by a combination of LiF and HCl; LiF + HCl etched products have fewer —OH and —F terminals and higher —O‐terminal content, which can provide higher capacity and facilitate the production of lithium‐ion batteries and other batteries with O‐terminal.^[^ ^150b^ ^]^ In addition to wet chemical etching of the MAX phase in a HF‐containing media, MXenes can also be synthesized from the Ti_3_AlC_2_ phase by utilizing halogens (Br_2_, I_2_, ICl, IBr) in an anhydrous medium. The surface composition can be effectively controlled by turning the molar ratio of halogen to the MAX phase, the absolute concentration of halogens, the solvent, and the temperature.^[^ ^158^ ^]^

MXenes with tunable surface terminations can also be made by molten salt etching.^[^ ^159^ ^]^ Through manipulating the phase transition of the selected salt melt and the temperature and composition of NaCl/ZnCl salt mixture, MXenes with optimal porosity and surface termination can be directly synthesized.^[^ ^160^ ^]^ Wang and co‐workers^[^ ^45^ ^]^ proposed a molten‐salt‐assisted‐electrochemical‐etching method to synthesize fluorine‐free TiC_2_Cl_2_. By adding various inorganic salts to in situ modify the surface terminals from —Cl to —O and —S, the obtained MXenes can be made into supercapacitor with excellent rate performance and capacitance retention. The alkali solution etches can also modify that surface groups by turning the concentration, the temperature, and the type of alkali solution. For example, the C–Ti–O ratio of MXenes treated with KOH increases from 22.12% to 49.69%, and the C–Ti–OH ratio decreases from 74.88% to 25.47%. With the increase of temperature, the Seebeck coefficient, as the thermoelectric figure of merit related to the electronic band structure of the material, increased due to the enhancement of the surface terminal modification effect, and the conductivity showed a downward trend.^[^ ^161^ ^]^

##### Matter Intercalation

Introducing low‐dimensional nanomaterials, ions, or molecules in the intermediate layer takes full advantage of the surface adsorption characteristics to modulate terminals.^[^ ^143^, ^145^, ^161^ ^]^ At the same time, intercalation is also an effective strategy to solve restacking arising from multilayer MXenes.^[^ ^145^, ^162^ ^]^ By a similar hydrothermal method using other reagents (such as NaOH, NH_4_OH, and KCl) as modification solvents, Na–Ti_3_C_2_T*_x_*, K–Ti_3_C_2_T*_x_*, and Cl–Ti_3_C_2_T*_x_* films showed higher electrical conductivity than NH_4_–Ti_3_C_2_T*_x_* films, which was mainly due to the strong adsorption force between the positive ions and the negatively charged MXene nanosheets. With the decreasing pH value of solvents, the Seebeck coefficient of modified MXene film decreases, indicating that the alkaline environment is conducive to converting surface functional groups. In the chemical intercalation of potassium salts, the —F termination is replaced by an oxygen‐containing functional group. Combined with the increase of the specific surface area of MXene film and the change of surface chemistry, the capacitance in sulfuric acid is increased by 4 times.^[^ ^163^ ^]^ The long‐chain fatty amine is inserted into the Ti_3_C_2_T*_x_* MXene interlayer by carbon‐intercalated, which can increase the interlayer spacing and remove the part of surface groups; the sandwiched structure facilitates electrolyte ions to penetrate the MXenes, and the interconnected network provides rapid ion/electron transfer.^[^ ^145^ ^]^ The results of intercalation and surface modification of the Ti_3_C_2_ monolayer show that the original monolayer acts as a magnetic metal, and the magnetic moment comes from Ti^2+^ on both sides.^[^ ^124^, ^143^ ^]^ The chemical doping of WS_2_ in the MXene nanoflake enables the regulation of the carrier density, and promotes the charge transfer between form disulfide to MXenes, thereby realizing the high photoluminescence yield of the atomic transition metal disulfide. A WS_2_/N–Ti_3_C_2_T*_x_* heterostructure junction for defluorination and elimination of adsorbed H_2_O was obtained by intercalation with ammonia at 700 °C, and the constructed MXene heterostructure junctions with controllable electron withdrawing ability offered a new avenue for the development of high‐performance photodetectors, ambipolar transfer field effect transistors, and flexible solar cells.^[^ ^155c^ ^]^

##### Heating and Oxidation Treatment

**Figure 8:**
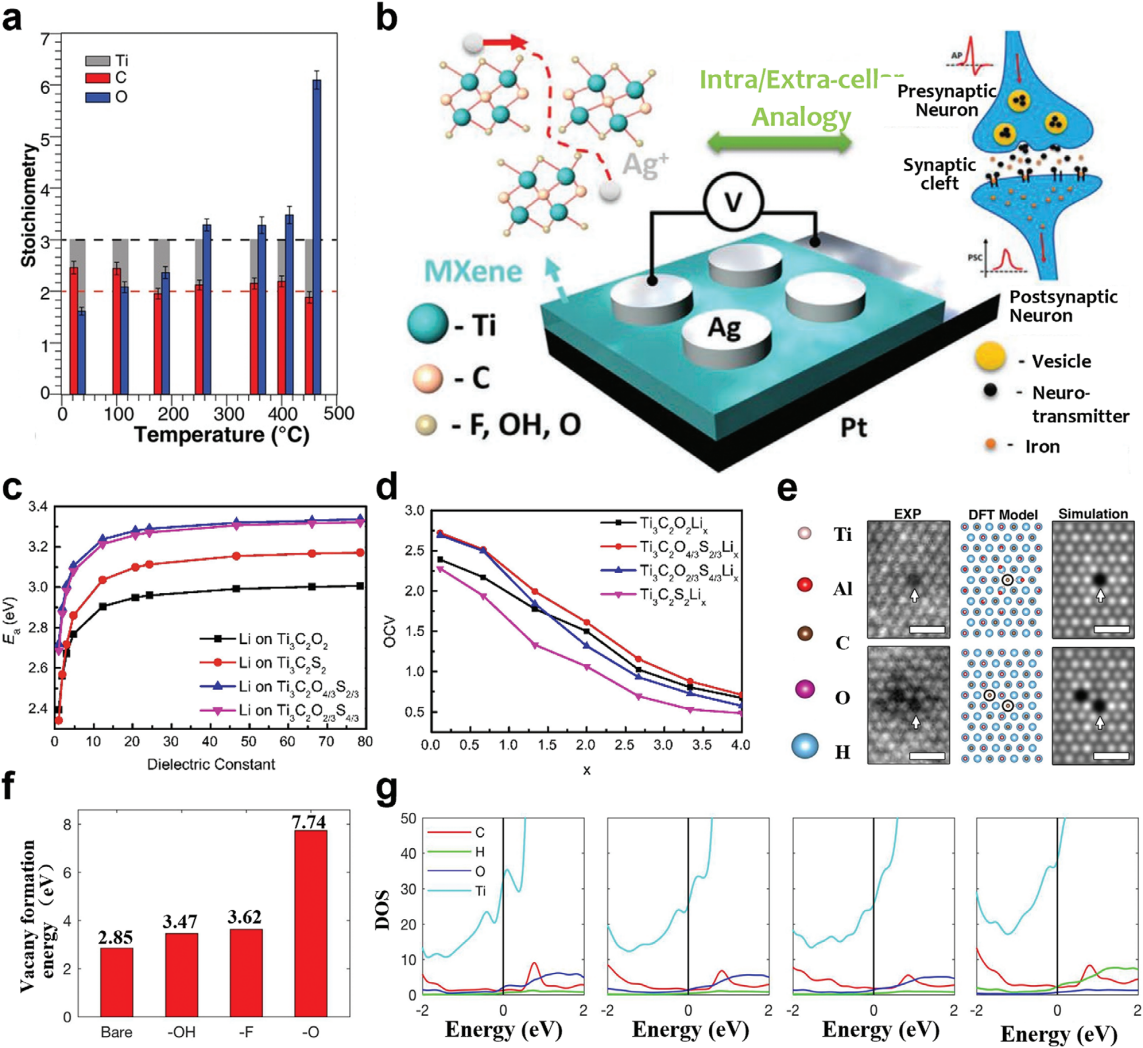
a) C/Ti/O stoichiometry quantitated by EELS from room temperature to 450 °C. Reproduced with permission.^[^ ^141b^ ^]^ Copyright 2020, Wiley‐VCH. b) The fabricated device in Ag/Ti_3_C_2_T*_x_* NS/Pt structure. Ag/Ti_3_C_2_T*_x_* NS/Pt device structure. Ag migration via MXene nanosheets are similar to ion migration in biological synapses. Reproduced with permission.^[^ ^167^ ^]^ Copyright 2020, Wiley‐VCH. c) The adsorption energies (*E*_a_) as a function of dielectric constant in Ti_3_C_2_T_2_ (T = O or S) and Ti_3_C_2_O*_y_* S_2−_ *_y_* (*y* = 4/3 and 2/3). d) The open‐circuit voltage (OCV) as a function of Ti_3_C_2_O_2_Li*_x_*, Ti_3_C_2_O_4/3_S_2/3_Li*_x_*, Ti_3_C_2_O_2/3_S_4/3_Li*_x_*, and Ti_3_C_2_S_2_Li*_x_*. Reproduced with permission.^[^ ^168a^ ^]^ Copyright 2020, Elsevier. e) Atomic‐scale high angle annular dark field‐Scanning transmission electron microscopy (HAADF‐STEM) images of defects in single‐layer Ti_3_C_2_T*_x_*. Comparison between experimental HAADF‐STEM image, defect crystal structure determined from DFT, and simulated HAADF‐STEM image of V_Ti_ and two adjacent V_Ti_ within the same sublayer. f) V_Ti_ formation energy on bare Ti_3_C_2_ and terminated single‐layer Ti_3_C_2_T*_x_*. g) Calculated DOS of —OH‐terminated Ti_3_C_2_ monolayer with pristine, V_Ti2_, V_Ti4_, and V_Ti6_ cluster surface (from left to right). Reproduced with permission.^[^ ^173^ ^]^ Copyright 2016, Royal Scoiety of Chemistry.

The composition and proportion of the surface terminals are highly sensitive to temperature and oxygen. The surface of MXenes undergoes defunctionalization after vacuum annealing.^[^ ^157^ ^]^ MXene exposure to oxygen also leads to supersaturation of oxygen on the surface and conversion to oxides.^[^ ^141b^ ^]^ Among the available surface groups, the F atom is easily desorbed under vacuum heating and the oxygen atom is inclined to be converted into oxides in an air environment.^[^ ^141^, ^164^ ^]^ As the temperature exceeds 550 °C, the —F terminal group is gradually desorbed and wholly removed at 750 °C. The existence of O‐functionalized surface could retain the highly ordered structure of MXenes after heating.^[^ ^165^ ^]^ As shown in **Figure** **8**a, at the initial high temperature treatment, hydrocarbons occupy the defluorinated vacancies. In addition, the surface of each Ti atom was covered with 0.8 oxygen atoms at the onset of the experiment. The oxygen‐terminal reached the supersaturated (*x* = 3.5) at 250 °C, and the Ti:O content on the surface was close to the oxygen content of TiO_2_ at 450 °C. The physisorbed water is desorbed after heating above 200 °C, but the water molecules were remained at 700 °C.^[^ ^141b^ ^]^

With the increase in temperature, the oxidation rate increased during the oxidation, and the oxidation rate was also affected by the oxidant according to the following rules: H_2_O_2_ > wet air > dry air.^[^ ^164^ ^]^ Water and dissolved oxygen in the suspension are the leading causes of oxidation for MXenes. Pinholes and defects in the synthesis process can also shorten the shelf life. For devices with high requirements on electrical conductivity, it is usually desirable to restore the intrinsic MXene property. The main methods to avoid oxidation are to store the materials in a low‐temperature vacuum environment,^[^ ^166^ ^]^ disperse them in nonaqueous solvents, and add salt for protection.^[^ ^59^, ^141^ ^]^ Sometimes oxidation or partial oxidation also plays a positive role. Using the unique migration dynamics of Ag^+^ cations via MXene insulating medium, Ag/Ti_3_C_2_T*_x_*/Pt device was constructed, as shown in Figure 8b. The migration of Ag^+^ cations is similar to Ca^2+^ irons dynamics of a biological synapse, and the device conductance could be effectively modulated.^[^ ^167^ ^]^

##### Chemical Modification

Chemical modification enables to install and remove surface groups more directly by performing substitution and elimination reactions. MXenes with surface terminations of bare, O, NH, S, Cl, I, Se, Br, and Te have been successfully synthesized in molten inorganic salts.^[^ ^153^, ^158^, ^160^ ^]^ Different surface groups have different interatomic distances in the MXene lattice. It has been found that the electronic transport properties of Nb_2_CT*_n_* with superconducting state have a strong dependence on the surface functional groups. The mixed —O‐, —OH‐, —F‐terminated Nb_2_CT*_n_* has two orders of magnitude higher resistivity and no superconductivity; the resistivity of Cl‐terminations begins to increase when Nb_2_CT*_n_* is cooled below 30 K. According to the difference in tunneling rate for charge carriers between MXene sheets due to localization, the resistivity of Nb_2_CT*_n_* MXenes terminated with the chalcogenide ion (Se < S < O) increases gradually when the sample is cooled.^[^ ^153^ ^]^ The theoretical calculation on the electrochemical performance of the Ti_3_C_2_T*_x_* model with different coverages of sulfur and oxygen functions has revealed that, with the synergistic effect of oxygen and sulfur functional groups, the adsorption of lithium atom on MXene surface becomes more stable. It is caused by the charge density difference and lattice constant calculations (Figure 8c,d). The adsorption energy with different proportions of O:S followed the order: Ti_3_C_2_O_4/3_S_2/3_ > Ti_3_C_2_O_2/3_S_4/3_ > Ti_3_C_2_S_2_ > Ti_3_C_2_O_2_.^[^ ^168^ ^]^

Other chemical groups can also be anchored onto the surface of MXenes. The physical and chemical bonds of free amine groups on the surface of MXenes can be achieved by using silane coupling agent, and the surface charge changed from −35 to +25 mV, which allows for the preparation of in situ self‐assembled film hydrolysis of the silane coupling agent named 3‐(2‐aminoethylamino)propyltrimethoxysilane (AEAPTMS). AEAPTMS releases methoxy groups in the form of methanol as the reaction side product.^[^ ^150d^ ^]^ The calculations by DFT and MD theory suggest that methoxy‐terminated MXenes can be stable.^[^ ^169^ ^]^ Through interfacial nucleophilic addition and sequential condensation reaction, alkylphosphonic acid ligand was grafted onto the hydroxyl terminal groups of the liquid–liquid interface of Ti_3_C_2_T*_x_* flake to form a covalent Ti—O—P bond at room temperature. It was demonstrated that surface‐functionalized Ti_3_C_2_T*_x_* MXene dispersions could be prepared by interfacial chemical grafting and phase transfer.^[^ ^170^ ^]^

#### 3.2.3 Targeted Amelioration of Defects

Defects are commonly determined on the surface of MXenes, which have an obviously impact on the periodic crystal structure and the surrounding charge distribution. Fully understanding the existence of defects and their mechanism will influence the investigation of the practical application of MXenes. The effects of point defects on the surface structure are explored with various detection techniques and DFT calculations.^[^ ^171^ ^]^ Calculation results show that the surface of the single‐layer structure is prone to point defects in the etching process because of the direct contact with the etching solution.^[^ ^172^ ^]^ As shown in Figure 8e, different Ti point defects (V_Ti_) observed in single‐layer Ti_3_C_2_T*_x_*, including single Ti vacancies (V_Ti_) and Ti vacancy clusters ($V_{\mathrm{Ti}}^{C}$), *C* equals the number of clustered vacancies, the defects concentration can be changed by adjusting the HF concentration in the etchant. The influence of different functional groups on the V_Ti_ formation energy will also determine the stability of the vacancy in each case. As shown in Figure 8f, the V_Ti_ formation energy order is: O>bare ≈ F. Similar results can also be observed in the defect formation of double metallic ordered Mo_2_TiC_2_T*_x_*, and calculations indicate that defects are more feasible in the outer Mo layers than in the inner Ti layers.^[^ ^172^ ^]^ Using ab initio molecular dynamics simulation, the bare monolayer with the defect on one side and pristine surface on the other side was immersed in HF for observation.^[^ ^173^ ^]^ The result suggests that water molecules are attached to the defective surface around Ti vacancies, while the water molecules on the pristine surface are fully dissociated. For vacancy clusters of $V_{\mathrm{Ti}}^{2}$ or larger, the protons and carbon atoms form C—H bonds in the defective. It is found that the Fermi level of the defective MXenes shifts downward resulting in DOS decreases at the Fermi level in the fully —OH‐terminated Ti_3_C_2_T*_x_* band diagram of Figure 8g. Interestingly, the calculated DOS indicates that the defective —OH‐terminated monolayer is still metallic. Therefore, defects can influence the surface morphology and terminal groups, but has little effect on the metallic conductivity. In the structure of the double sheets, high oxygen saturation is observed between the bilayer films, and apparent mobile of surface clusters and intrinsic defects is observed.^[^ ^174^ ^]^

The adsorption group on the surface of MXenes can effectively modify V_Ti_. In the Li‐ion battery structure, the Al_2_O_3_ nanoclusters anchored at the V_Ti_ sites can improve the ion diffusion kinetics on the surface of MXenes and alleviate the irreversible electrolyte decomposition and ion dendrite formation tendency caused by the defects.^[^ ^175^ ^]^ Besides, CO_2_ molecules can be adsorbed on the defect surface of Mo_2_TiC_2_T*_x_* during the spontaneous exothermic process.^[^ ^176^ ^]^ MXenes can also be taken as an electrocatalyst for the electrochemical carbon dioxide reduction reaction. When V_Ti_ is present on most MXenes, the Fermi energy level is shifted and the C‐coordinated fragment‐type intermediates (—COOH, —CHO) become more strongly bond, while the H‐coordinated molecular intermediates (HCOOH, H_2_CO) are not affected. The defective Hf_2_NO_2_ MXenes containing Hf vacancies have a low potential of 0.45 V.^[^ ^177^ ^]^ Thus, defects have a significant influence on the physical and chemical properties of the electrode material. It allows for flexible control of material application performance by constructing building defects, such as a plasma‐assisted mechanical chemical method. After the plasma‐assisted mechanical chemical treatment, the layered structure of TiC_2_T*_x_* MXenes is distorted, and numerous defects act as additional active sites of lithium ions, so that the electrochemical energy storage capacity of the lithium‐ion battery is enhanced.^[^ ^178^ ^]^ In the same way, Mo vacancies with uniform distribution are constructed in the composite material Mo*_x_* S/TiO_2_/Ti_3_C_2_, and the introduction of Mo vacancies inhibits the recombination of carrier recombination and increases plentiful active sites for photocatalytic hydrogen production.^[^ ^179^ ^]^ Layered TiO_2_ oxidized from Ti_3_C_2_T*_x_* was used as a catalyst for the synthesis of ethylene (M–TiO_2_). Ti on M–TiO_2_ can stabilize the defective structure, and oxygen vacancies are conducive to the activation of O_2_.^[^ ^171a^ ^]^ In addition to single transition metal MXenes, double transition‐metal MXenes also have defect modification effects. Zr, Mo, Hf, Ta, W, Re, and Os supported on defective Mo_2_TiC_2_O_2_ MXenes can significantly promote the nitrogen reduction reaction, and the Zr‐doped atoms catalyst has the lowest potential barrier (0.15 eV).^[^ ^176^ ^]^

### 3.3 Contact Principle of MXenes with Semiconductor

**Figure 9:**
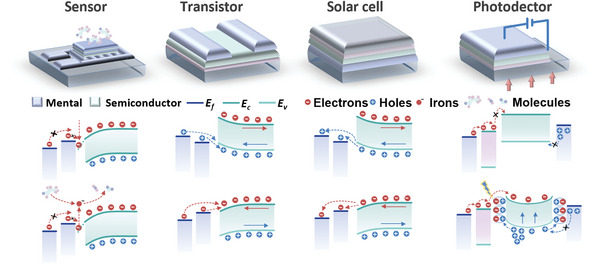
Interfacial contacts and charge carrier behaviors in electronic devices.

The contact between metal and semiconductor exists in various electronic devices, and the state of contact interface, energy level matching, and energy band turning are crucial for balancing the different performance indicators of devices. **Figure** **9** lists the charge transfer behaviors (including injection, collection, transportation) in electronic devices. The design of materials and structures can be effectively guided by an in‐depth understanding of the contact principle and modulation method. This section expounds the contact mechanism, modulation means, and contact types between MXene electrode and semiconductor from the aspects of Schottky barrier, Fermi pinning effect, and work function at the interface.

#### 3.3.1 Contact Barrier

The interface formed at the point of contact between metal electrodes and semiconductor material is a crucial component of modern electronic and optoelectronic devices, passing an energy barrier for the charge transport process known as potential barrier.^[^ ^180^ ^]^ Ideally, metal and semiconductor are separated from each other at infinity and independent from each other before contact, so they have a common vacuum energy level. Their energy band structures include Fermi energy level and work function. The difference between the potential (*V*_m_) on the metal side and the semiconductor surface potential (*V*_s_) is defined as the contact potential difference (*V*_m_ − *V*_s_ < 0). The specific processes are as follows: when the metal is far away from the semiconductor, the induced charge generated by the semiconductor is very little, the electric field is very weak, and the energy band structure is not bent; as the two approaches gradually, the distance becomes shorter and the electric field strength is enhanced. When they are completely close, the contact potential difference all falls on the semiconductor, resulting in the bending of the semiconductor energy band at the end.^[^ ^181^ ^]^ For electrons, the bending of the energy band is the same as that of p–n junction, forming a potential barrier (*qV*_D_ = *V*_m_ − *V*_s_); for metals, forming Schottky Barrier (*qΦ*_nS_ = *W*_m_ − *χ*), where *W*_m_ is the work function of the metal. A simplified energy band diagram of that Schottky barrier formed by the metal‐semiconductor contact is shown in Figure 4.

When *W*_m_ > *W*_s_, the Fermi energy level of the metal is low, and the electrons in the semiconductor flow to the metal, where *W*_s_ is the work function of the semiconductor. After the contact is balanced, there is a lack of electrons in the semiconductor, and the bottom of the conduction band near the metal region is further away from the Fermi energy level. Compared with that in semiconductor interior, the concentration of carriers on the side near the metal is lower, forming a potential barrier. The potential barrier region is high‐resistive for electron transmission, and has certain rectifying characteristics, so it is called a barrier layer.^[^ ^182^ ^]^ When *W*_m_ < *W*_s_, metal electrons continuously fly to the semiconductor until they reach equilibrium, and electrons on the surface of the semiconductor are in surplus. Moreover, the bottom of the conduction band at one side of the metal is close to Fermi energy level or even lower than Fermi energy level, resulting in the merger and downward bending of the energy band. At this moment, no matter in which direction the electrons are transmitted, no potential barrier is generated. Since a large number of electrons are attached to the surface, the surface becomes a low resistance layer, and an Ohmic contact is formed. Barrier and antibarrier layers, Schottky and Ohmic contacts can also be formed for p‐type semiconductors.^[^ ^183^ ^]^

Several states of metal–semiconductor contact are extensively present in various semiconductor devices. Regarding the contact principle, the strong binding of most electrode materials in direct contact with the semiconductor leads to the pinning effect, which hinders carrier transport. It will be described in detail in the next section. By eliminating the pinning effect, the potential barrier can be more precisely predicted by the intrinsic properties of metals and semiconductors. One way to improve charge transport is substitutional doping^[^ ^184^ ^]^ or inserting a thin buffer layer between the metal and semiconductor.^[^ ^185^ ^]^ However, it is not suitable for all manufacturing conditions and easily modifies the 2D material and its properties. Another way is to use 2D metal substitute for ordinary electrode to suppress the pinning effect. The 2D metal surface has no dangling surface bonds compared to 3D metal, and thus the contact with the 2D semiconductor is more prone to the formation of van der Waals contact. Moreover, 2D materials exhibit better gate electrostatics than bulk electrodes due to the weaker electric field shielding effect.^[^ ^186^ ^]^ MXenes as a 2D material, the transition metal elements of d‐electrons can overlap with 2D semiconductor materials of d‐electrons,^[^ ^187^ ^]^ and different combinations of elements and functional groups impart work function that can be adjusted in an extensive range resulting in better charge injection.

#### 3.3.2 Fermi Level Pinning

The Schottky barrier of the metal/semiconductor contact can be predicted by the Equations (6) and (7)

(6) $$\Phi_{\mathrm{SB},n}=W_{m}-\chi_{S}$$

(7) $$\Phi_{\mathrm{SB},p}=I_{S}-W_{m}$$

*Φ*_SB,n_, *Φ*_SB,p_ are the Schottky barrier heights (SBH) of electrons and holes, respectively. *χ*_S_ and *I*_S_ are the electron affinity and ionization potential of semiconductors, respectively.^[^ ^188^ ^]^ Due to the *χ* and *I* intrinsic properties of the semiconductor material determined before contact, the Schottky barrier and the work function have linear relationships. However, the SBH does not absolutely obey the work function. The surface state in semiconductor layer can fix the Fermi level in the semiconductor bandgap, which is called the Fermi level pinning effect (FLP). The strength of the pinning effect is expressed by the interface *S* parameter

(8) $$S=\left|d\varphi_{\mathrm{SB}}/dW_{m}\right|$$

When *S* = 1, the ideal Schottky–Mott model is established. However, the *S* of most semiconductors is typically less than 1.^[^ ^184^, ^188^ ^]^ The linear relationship between SBH and work function (*W*_s_) can be destroyed by that pinning effect for different causes in the contact system, causing a high contact resistance that hinders the realization of Ohmic contact.

**Figure 10:**
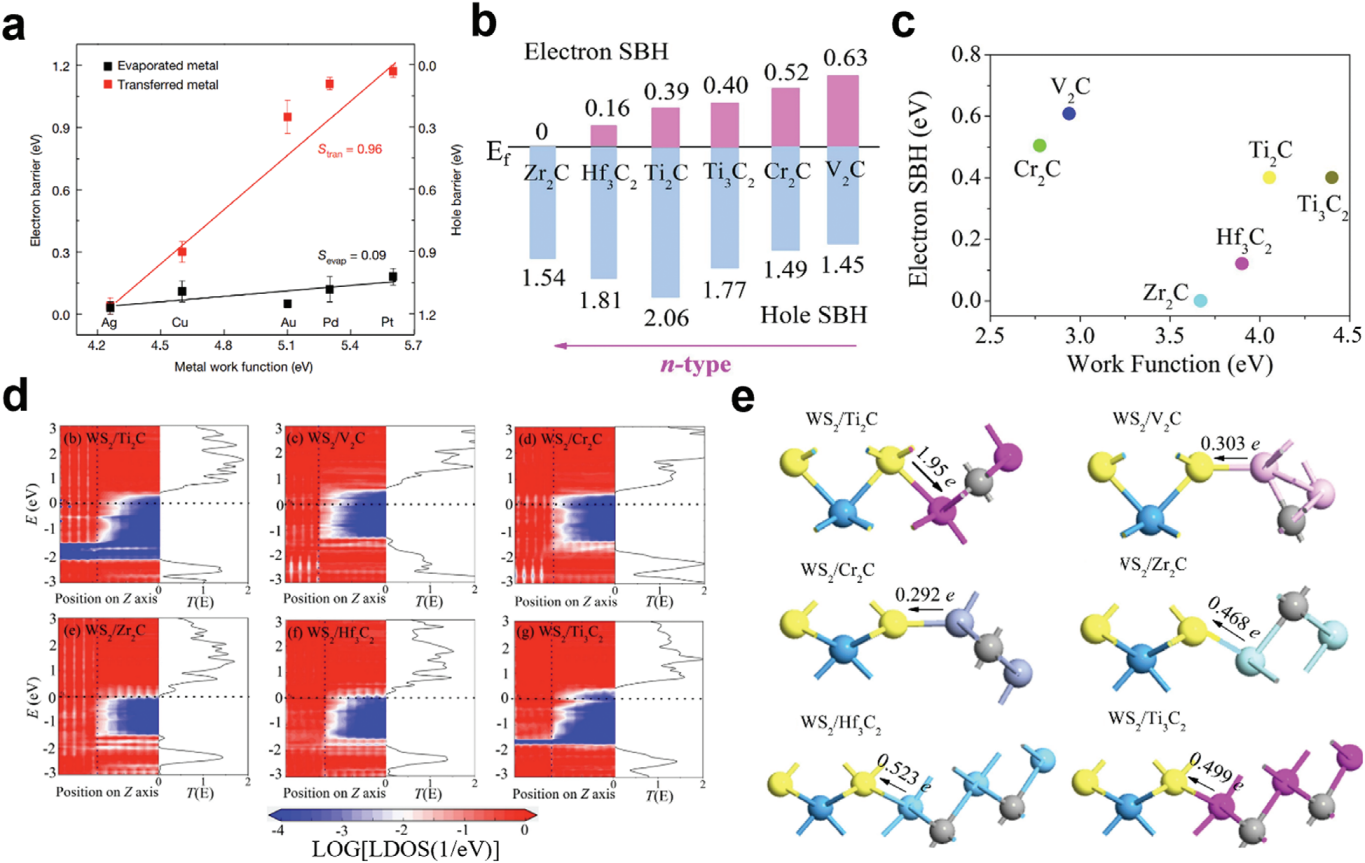
a) Schottky barrier heights for different transfer and evaporation metals. Reproduced with permission.^[^ ^188a^ ^]^ Copyright 2018, Springer Nature. b) Schotty barriers heights (SBH) of electron and hole carriers for monolayer WS_2_ with various MXene electrodes. c) Electron SBH of MXene electrode material as a function of work function. d) The local density of state (LDOS) projected to the position of the monolayer WS_2_ layer and transmission spectra of monolayer WS_2_ transistors with various MXene electrodes. d) Electron SBH of MXene electrode material as a function of work function. e) Bader charges for WS_2_/Ti_2_C; WS_2_/V_2_C; WS_2_/Cr_2_C; WS_2_/Zr_2_C; WS_2_/Hf_3_C_2_; and WS_2_/Ti_3_C_2_. Reproduced with permission.^[^ ^195^ ^]^ Copyright 2020, Elsevier.

The traditional device manufacturing process usually leads to the appearance of surface chemical disorders,^[^ ^189^ ^]^ metal‐induced gap states (MIGS)^[^ ^190^ ^]^ and defect/disorder‐induced‐gap states (DIGS).^[^ ^191^ ^]^ For example, typical metal deposition processes involving bombardment of atoms or clusters and intense local heating of contact areas can damage the lattice arrangement at and around the interface. The resulting interfacial contact is commonly a chemical bond with a strong interaction.^[^ ^192^ ^]^ Second, the use of resist also leaves polymer residue at the interface, causing the barrier height of the overall test to deviate from the predicted value.^[^ ^193^ ^]^ Finally, the metal wavefunction decays and penetrates into the semiconductor to form a metal‐induced gap state. All of the above are easy to induce the Fermi pinning effect.^[^ ^194^ ^]^ Because the deposition process of printing method avoids high temperature and chemical bonding to a greater extent, it tends to form vdW contact interface. Studies have shown that a vdW metal semiconductor heterojunction is fabricated by transfer printing. The extracted *S* is 0.96 in the relationship between the potential barrier and the work function of the electrode, while that of the electrode prepared by deposition is 0.09 (**Figure** **10**a). This indicates that the heterojunction forms a close to ideal Schottky–Mott contact.^[^ ^188a^ ^]^

It is found that in the WS_2_/MXene contact system, the lateral Schottky barrier has a linear relationship with the *W*_F_ of the metal electrode. However, the contact barrier is not a linear relationship in actual contact (Figure 10b,c). According to the analysis of the Bader charge and band diagram, the stronger coupled contact between WS_2_/TiC_2_ resulted in severe metallization of WS_2_ and abnormal FLP effect. On the other hand, moderate coupling strengths were observed between WS_2_ and MXenes Zr_2_C, Ti_3_C_2_, and Hf_3_C_2_. Intense charge transfer from Zr_2_C, Ti_3_C_2_, and Hf_3_C_2_ to WS_2_ forms interface dipole. These interface dipoles facilitate the conduction band minimum (CBM) of WS_2_ to move to the Fermi level, resulting in a decrease in the lateral Schottky barrier electron carriers. For both WS_2_/V_2_C and WS_2_/Cr_2_C systems, a weak interfacial dipole effect is observed due to the weak charge transfer from the MXene electrode to WS_2_ and the high lateral Schottky barrier of the electron carriers. Therefore, ideal Ohmic contact in that vertical and lateral direction can be formed in WS_2_/Zr_2_C and WS_2_/Hf_3_C_2_ transistors which have higher electron injection efficiency than transistors based on Ti_2_C, V_2_C, Cr_2_C, and Ti_3_C_2_ electrodes (Figure 10d,e).^[^ ^195^ ^]^

#### 3.3.3 Work Function

**Figure 11:**
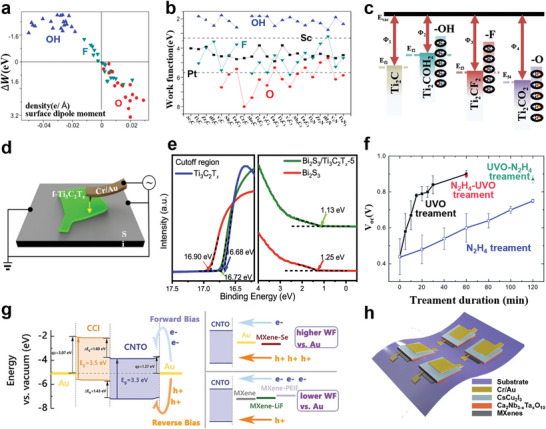
a) The work function change caused by the surface termination is a function of the surface dipole moment density. Inset is a schematic diagram of the surface dipole effect. b) The working function of MXenes with various terminals. Bare surface, black square; —O termination, red circle; OH, blue up‐triangle; F, cyan down‐triangle. For comparison, the work functions of the metals Sc and Pt are shown in dashed lines. Reproduced with permission.^[^ ^4b^ ^]^ Copyright 2016, American Chemical Society. c) Dipole lay formation induced by OH, F, and O and interfacial dipole‐induced Ti_2_C *E*_f_ shift (*E*_vac_ indicates the vacuum level; *E*_f_ indicates the Fermi level; *Φ* indicates the work function). d) Schematic diagram of the Kelvin probe force microscope. Reproduced with permission.^[^ ^2b^ ^]^ Copyright 2021, American Chemical Society. e) UPS spectra measured by He I (*hν* = 21.22 eV) spectra the secondary electron cutoff of Ti_3_C_2_T*_x_* and valence band of Bi_2_S_3_ and Bi_2_S_3_/Ti_3_C_2_T*_x_*_−5_ Schottky catalyst with respect to the Fermi level (*E*_f_). Reproduced with permission.^[^ ^200^ ^]^ Copyright 2021, Springer Nature. f) *V*_oc_ varies with the UVO, N_2_H_4_, N_2_H_4_–UVO, and N_2_H_4_–UVO treated duration.^[^ ^199^ ^]^ Copyright 2019, Royal Scoiety of Chemistry. g) Energy band diagram of Au/p‐CsCu_2_I_3_/n‐Ca_2_Nb_3−_ *_x_* Ta*_x_* O_10_/MXene device. h) Schematic of Au/p‐CsCu_2_I_3_/n‐Ca_2_Nb_3−_ *_x_* Ta*_x_* O_10_/MXene device with different MXenes as bottom electrodes. Reproduced with permission.^[^ ^104^ ^]^ Copyright 2022, Wiley‐VCH.

The *W*_F_ is defined as the minimum energy required to move an electron from the interior of a solid to the surface of an object. The work function of MXenes can be expressed by the energy difference between the Fermi level and the vacuum level. In actual contact, the Fermi levels are related to work functions but not equal. They are highly sensitive to changes in material structure and composition, and surface physical and chemical properties. Compared with the bare MXenes, the existence of surface functional groups induces the surface effect yielding the distribution of interface dipoles to generate surface dipole moments,^[^ ^4b^ ^]^ which are affected by the redistribution of electrons, surface relaxation caused by the groups, and the polarity of the group^[^ ^3^, ^196^ ^]^ (**Figure** **11**a).

The work function can be expressed as ∆*φ* = −(*e*/*ε*_0_)∆*P*, where *e* is the amount of electron charge, *ε*_0_ is the vacuum dielectric constant, and *P* is the surface dipole moment. The slope in the equation is calculated to be about 170.012 V Å, indicating that the work function is linearly related to the surface dipole moment. It is found that the work function fluctuates the most with different types and proportions of functional groups on the surface^[^ ^3^, ^4^, ^196^, ^197^ ^]^ (Figure 11b). The work function of MXenes has been calculated to range from 1.8 to 8.0 eV (Sc_2_C(OH)_2_–Cr_2_CO_2_) with a semiconducting MXene bandgap in the range of 0.04–3.23 eV.^[^ ^3^, ^4^, ^196^ ^]^ Using first‐principle calculations based on the DFT, Xin and Yu^[^ ^4a^ ^]^ proposed that the work function of methoxy‐ionized niobium carbide Nb_3_C_2_(OCH_3_)_2_ MXenes can reach as low as 0.9 eV due to the hybridization of the transition metal d‐orbital and O 2p‐orbital. However, this ultralow work function has not yet been experimentally verified, thus pointing out a challenging issue for further studies.

There are three main reasons for the difference in work function between the intrinsic MXenes. i) The difference in surface dipole moment is caused by different types and numbers of surface functional groups (Figure 11c). The —F‐ and —O‐terminated changes in the total dipole moment are due to the combination of two sides. The first is the redistribution of the transferred electron charges between the substrate and the functional groups, and another is the change in the dipole moment due to surface relaxation.^[^ ^196^ ^]^ ii) The differences of SOC effects between different MXenes—the transition metals with heavy 4d and 5d orbitals have a stronger SOC effect.^[^ ^140^ ^]^ In general, SOC further increases valence band maximum (VBM) and reduces CBM. For 2D transition metal dichalcogenides, the SOC effect is negligible.^[^ ^4^ ^]^ iii) Layer thickness—for the thin layer MXenes, it is susceptible to the quantum effect. Multilayer MXenes are vulnerable to interlayer interaction. Calculation results show that the bandgap of monolayer Ti_2_CO_2_ is 0.26 eV, which decreases to 0.0369 eV in the five‐layer configuration.^[^ ^198^ ^]^ iv) Differences in measurement techniques—currently, the work function of MXenes is mainly measured by the Kelvin probe force microscopy (KPFM) (Figure 11d) and the Ultroviolet Photoelectron Spectrometer (UPS) (Figure 11e).

Various experimental approaches, such as strain, vacuum annealing, intercalation adsorption, chemical doping, and ultraviolet ozone (UVO) treatment have been applied to tune the work functions of MXenes. Figure 4 shows the approaches used for regulating the work function of MXenes. It has been shown that the energy band structure of multilayer TiCO_2_ undergoes the transition from semiconductor to semimetal to semiconductor to metal.^[^ ^198^ ^]^ Koch and co‐workers^[^ ^106^ ^]^ investigated the effect of the different annealing temperatures on the electronic properties of Ti_3_C_2_T*_x_*. As annealing temperature and time were increased, the ratio of —O‐terminated and —F‐terminated on the Ti_3_C_2_T*_x_* surface increased slightly, the work function is increased by desorption of hydroxyl, water, as well as carbon‐dominated contaminations, and decreased by the fluorine desorption at high temperatures. Recently, blending Ti_3_C_2_T*_x_* with DDAB has been used to turn the properties of MXenes, resulting in the reduction of work function.^[^ ^109^ ^]^ In addition to physical modification, chemical modifications are often applied. The TiC_2_T*_x_* was modified by diazonium covalent chemistry. The work function is modifiable by regulating the quantity of grafted diazonium surface groups.^[^ ^2b^ ^]^ The chemical doping of NH_3_ can also expediently regulate the work function of MXenes and reduce the charge injection barrier of n‐type Organic Field‐Effect Transistor (OFET).^[^ ^107^ ^]^ Ouyang and co‐workers^[^ ^199^ ^]^ found that the work function of the spin‐coated MXene film could be decreased by placing it in a sealed Petri dish of N_2_H_4_ vapor (N–MXene). When MXene is treated by the UV ozone treatment (H–MXene) carried out in UV‐ozone cleaner, its work function is lowered. Further treatment of U–MXenes with N_2_H_4_ lowered the work function and endowed MXenes with reverse performance, which can act as an electronic transmission channel. As shown in the Figure 11f, *V*_oc_ varies in solar cell with the treatment duration, so the effective control of the electron/hole collecting layer can be realized by adjusting the order and the duration of the two treatments. Recently, the surface of MXenes film was coated with LiF and Se through thermal evaporation, and polyethyleneimine ethoxylated (PEIE) and MXenes were mixed to realize surface modification. The work function of MXenes could be controlled within the range of 4.55–5.25 eV.^[^ ^104^ ^]^ From the energy band diagram, it can be seen that PEIE‐modified MXenes and n‐type semiconductor Ca_2_Nb_3−_ *_x_* Ta*_x_* O_10_ formed a lower carrier transport potential barrier (Figure 11g). The rectification characteristics of the p–n junction photodetector constructed based on this structure were significantly enhanced (Figure 11h).

#### 3.3.4 Heterostructures

The contact type in metal–semiconductor contact can be predicted by the work function of the contact system energy band distribution. However, for many heterostructures, it still cannot be used as the only criterion due to the existence of FLP. Calculation results show that the weak vdW interaction weakens the FLP by reducing the surface state, and can effectively regulate SBH by forming contact systems with different work function matches.^[^ ^184^, ^201^ ^]^ Duan and co‐workers^[^ ^188^ ^]^ proposed a vdW heterojunction in which metal electrodes were prefabricated and physically laminated to a suspended, bond‐free, 2D semiconductor, avoiding direct chemical bonding.

Besides, MXenes can also form vdW heterostructure with WSe_2_, MoS_2_, graphene, blue phosphorus, black phosphorus, and others. The specific similar calculation parameters are shown in **Table** **3**. The binding energy (*E*_b_) and lattice mismatch indicate that the internal binding force of these heterojunctions is vdW. The larger *E*_b_ and smaller lattice mismatch indicate the more stable heterostructure. Overall, functionalized MXenes overall show better stability than bare MXenes by saturating the nonbonded valence electrons of the surface metal atoms.

**Table 3:** Calculated X/MXene vdW properties, including work function of X, MXenes, X/MXene structures, lattice mismatch, binding energy (*E*_b_), electronic/hole barrier (eV), tunneling barrier height (*φ*_T_). In the work function column, *E*_c_, *E*_v_, and *E*_f_ stand for conduction band minimum, valance band maximum, fermi level, respectively. In the binding energy column, there is a mark “*” and “**” in the binding energy to represent the unit as “J m^−2^” and “eV Å^−2^.” “X” represents material forming the vdW heterostructure with MXenes. “Oh” represents an Ohmic contact, “LS” represents a low Schottky barrier contact

Contact material	Work function	Contact	Work function [eV]	Mismatch [%]	Binding energy *E*_b_ [meV]	Heterojunction work function	Electronic barrier [eV]		Hole barrier [eV]		Tunneling barrier (*φ*_T_)		Ref.
							Vertical direction ($\varphi_{V}^{e}$)	Lateral direction ($\varphi_{L}^{e}$)	Vertical direction ($\varphi_{V}^{h}$)	Lateral direction ($\varphi_{L}^{h}$)			
ML WSe_2_	–	NbSe_2_	–	–	–	–	–	–	0	0	0		[20]
		Ti_2_C	–	–	–	–	0	0.43	0	–	0		
		Ti_2_C(OH)_2_	–	–	–	–	0	0	–	–	0		
		Mo_2_CF_2_	–	–	–	–	–	–	0	0	0		
		Mo_2_CO_2_	–	–	–	–	–	–	0	960	0		
2L WSe_2_		Ti_2_C	–	2.16	−120	–	0	0.40	0	0.68	0		[26]
		Ti_2_C(OH)_2_	–	2.18	−330	–	−0.04	−0.03	1.30	1.25	0		
		Mo_2_CF_2_	–	2.37	−120	–	1.25	1.25	0.02	0.02	0		
MoS_2_	*E*_c_: 5.97	Nb_2_C(OH)_2_	2.14	1	–	–	−0.03		1.69		–		[26]
		F	4.36	–	–	–	0.49		1.14		–		
		O	5.71	–	–	–	1.44		0.26		–		
C_2_N	*E*_c_: 4.42 *E*_v_: 6.08	Hf_2_C(OH)_2_	2.26	1.65	–	–	−0.21		1.65		–		[204]
		Nb_2_CF_2_	4.47	0.77	–	–	0.06		1.60		–		
		MO_2_NO_2_	5.82	0.61	–	–	1.34		0.33		–		
ReS_2_	4.58	Ti_3_C_2_	4.40	4.04	780	4.32	0	−0.15	0		1.20	0.24	[205]
	4.40	Hf_3_C_2_	4.58	0.13	740	4.53	0	−0.07	0		1.22	0.23	
InSe	*E*_c_: 4.37 *E*_v_: 6.51	TaSe_2_	5.65	−1.55	0.29*	5.55	0.86 (1L)		0.65 (2L)		2.20		[184]
		VSe_2_	5.75	−5.52	0.31*	5.64	0.57		0.46		2.38		
		NbSe_2_	5.82	−0.98	0.30*	5.74	0.58		0.40		2.15		
		TaS_2_	6.23	5.24	0.30*	6.13	0.28		0.10		2.40		
		VS_2_	6.30	−3.03	0.33*	6.29	0.39		–		2.16		
		NbS_2_	6.44	−4.67	0.33*	6.29	0.22		0.09		2.41		
MoS_2_	5.95	V_2_CO_2_	6.70	<1	0.018**	–	–		0.052		–		[187]
		Cr_2_CO_2_	7.65	<1	0.025**	–	–		0.005		–		
		Mo_2_CO_2_	7.50	<1	0.024**	–	–		−0.185		–		
		V_4_C_3_O_2_	6.65	<1	0.015**	–	–		0.027		–		
		Cr_2_NO_2_	7.00	<1	0.019**	–	–		−0.102		–		
		V_2_NO_2_	6.30	<1	0.016**	–	–		0.022		–		
MoS_2_	–	Ta_2_C	–	–	–	–	S		–		–		[27]
		Ta_2_CF_2_	–	–	–	–	Oh		–		–		
		Ta_2_C(OH)_2_	–	–	–	–	Oh		–		–		
Black Phosphorus	*E*_c_: 4.12 *E*_f_: 4.75 *E*_v_: 5.03	Zr_2_CO_2_	5.07	0.27	−160	–	–	–	–		–		[28]
		Zr_2_CF_2_	3.39	2.04	−140	4.30	Oh	LS	–		3.69		
		Zr_2_CO_2_H_2_	2.10	0.27	−380	4.26	Oh	LS	–		2.02		
		Zr_3_C_2_O_2_	5.13	0.26	−170	5.25	Oh	Oh	–		4.57		
		Zr_3_C_2_F_2_	3.65	0.37	−140	5.02	Oh	LS	–		4.09		
		Zr_3_C_2_O_2_H_2_	2.04	0.37	−350	4.18	Oh	LS	–		2.30		
Blue P	–	Ti_3_C_2_O_2_	–	4.30	−235.1	–	1.30		0.00		–		[29]
		Ti_3_C_2_(OH)_2_	–	2.70	−560.2	–	−0.17		1.70		–		
		Ti_3_C_2_F_2_	–	3.11	−254.2	–	1.34		0.68		–		
		Zr_3_C_2_O_2_	–	0.78	−81.5	–	1.45		−0.37		–		
		Zr_3_C_2_(OH)_2_	–	1.40	−630.5	–	0.00		1.65		–		
		Zr_3_C_2_F_2_	–	2.81	−476.9	–	−0.24		1.39		–		
		Hf_3_C_2_O_2_	–	0.02	−435.2	–	1.47		0.00		–		
		Hf_3_C_2_(OH)_2_	–	0.61	−431.8	–	−0.06		1.87		–		
		Hf_3_C_2_F_2_	–	0.52	254.2	–	0.12		1.75		–		
MoS_2_	–	Ti_3_C_2_O_2_	–	3.58	−185.6	–	1.70		−0.04		–		[240]
		Ti_3_C_2_(OH)_2_	–	1.99	−265.9	–	0.00		1.40		–		
		Ti_3_C_2_F_2_	–	2.39	−190.8	–	0.82		0.86		–		
		Zr_3_C_2_O_2_	–	1.45	−432.3	–	0.62		−0.23		–		
		Zr_3_C_2_(OH)_2_	–	2.06	−442.7	–	−0.25		0.75		–		
		Zr_3_C_2_F_2_	–	3.46	−328.6	–	−0.05		0.59		–		
		Hf_3_C_2_O_2_	–	0.66	−308.6	–	0.95		0.00		–		
		Hf_3_C_2_(OH)_2_	–	1.28	−434.8	–	−0.02		1.12		–		
		Hf_3_C_2_F_2_	–	1.19	−298.7	–	0.08		1.09		–		
Graphene	–	Cr_2_C	4.54	3.34	334	4.26	0	0.04	0		0.10	–	[241]
		Ta_2_C	4.94	3.34	334	4.63	0	0.01	0		0.02	–	
		V_2_C	4.80	3.34	334	4.46	0	0.00	0		0.20	–	
Tl_2_O	*E*_c_: 3.61 *E*_f_: 4.59 *E*_c_: 4.61	Ti_2_C	4.43	1.51	129	3.98	–	0.36	–		0.65	0	[203]
		Ti_2_C(OH)_2_	1.66	0.56	430	1.10	–	0.00	–		1.02	0	
		Ti_2_CF_2_	4.92	1.10	240	4.81	–	0.98	–		0.00	0	
carbon sulfide (CS)	–	Ti_2_CO_2_	–	–	−1234	–	0.58		0.69		–		[242]
		Zr_2_CO_2_	–	–	−39	–	0.50		2.59		–		
		Hf_2_CO_2_	–	–	−357	–	0.21		3.69		–		

Compared with the traditional metal–semiconductor junction contact, the contact system formed by vdW can inhibit DIGS and MIGS.^[^ ^184^, ^188^ ^]^ The two gap states act as a reservoir of electrons and holes and fix the Fermi level. Chemical bond contact is also prone to charge scattering and reduces carrier mobility. The forming of vdW contacts can reduce the effect of the increase in local density of states, whereas retaining the intrinsic energy band structure and electronic properties of the contact system to the greatest extent. Another advantage of forming weak vdW contacts is that they provide more flexible band structure selectivity, and different contact systems are constructed by selecting different transition metal types, surface functional groups, and layer numbers to satisfy the application in multilayer devices.

Table 3 also shows that the energy band arrangement of the MXene‐based heterostructure is different from the respective intrinsic energy band arrangement of the contact materials. The electronic state near the Fermi level is susceptible to the heterogeneous interface, enabling the SBH of the heterojunction to be adjusted by interface engineering. For example, the work function of the hydroxyl‐terminated MXenes is larger than that of the 2D GaN, it is predicted that Schottky contact should be formed.^[^ ^202^ ^]^ In fact, they form an Ohmic contact, and the intrinsic polarization of the GaN nanosheets and the surface functional groups of MXenes have a great effect on the interfacial charge transfer and depolarization field formation of MXene/GaN heterojunction. By calculating the SBH and tunneling barrier, it can be predicted that GaN/Hf_2_C(OH)_2_ and GaN/Zr_2_N(OH)_2_ are expected to form the ideal Ohmic contact.^[^ ^202a^ ^]^ Similarly, Tl_2_O forms n‐type Ohmic contact with the Ti_2_C(OH)_2_ electrode, p‐type Ohmic contact with the Ti_2_CF_2_ electrode, and Schottky barrier with the bare Ti_2_C.^[^ ^203^ ^]^

Quhe and co‐workers^[^ ^20b^ ^]^ performed calculations of the interface properties and device performance of WSe_2_ transistors, by combining DFT and PBE‐GGA functional approaches, suggesting a high‐performance, low‐power‐consumption transistor with short delay time and low on‐current was constructed based on NbSe_2_ and Ti_2_(OH)_2_. They proposed the regulation of van der Waals interface interaction, the Schottky and tunneling barrier, and the MIGS. In particular, the low doping of the channel is crucial for the realization of Ohmic contact or low Schottky contact devices.

Appropriate surface functionalization and external electric field can also effectively turn the SBH of single‐layer MoS_2_/MXene heterostructure to maximize the retention of the intrinsic energy band structures of the two materials. They are combined through vdW. According to the calculation and prediction results, Yu and co‐workers^[^ ^187b^ ^]^ synthesized the MoS_2_/Nb_2_CT*_x_* heterostructure by hydrothermal method for the first time. Both the theoretical calculation and experimental results show that the bottom p‐type contact improves the electrocatalytic performance of the heterostructure by improving the charge conductivity, charge transfer speed, and hydrogen adsorption capacity. Similarly, monolayer C_2_N can also form a vdW contact heterojunction with MXenes (Hf_2_C(OH)_2_ Nb_2_CF_2_, MO_2_NO_2_).^[^ ^204^ ^]^ ReS_2_/Ti_3_C_2_, ReS_2_/Hf_3_C_2_ can also induce the formation of Ohmic contact due to their strong interaction and low work functions relative to silicon, boron, aluminum, nickel, and copper metals.^[^ ^205^ ^]^ In addition, other results reveal that the transition from Schottky contact to Ohmic contact can also be achieved by choosing the appropriate layer number. CBM increases and VBM decreases because of the increase of the InSe layer number. By comparing among 2D metals (M_3_C_2_ (M = Cd, Hg, Zn); XA_2_ (X = Ta, V, Nb; A = S, Se)), Cd_3_C_2_ would be the best electrode candidate for bilayer InSe.^[^ ^184b^ ^]^

**Figure 12:**
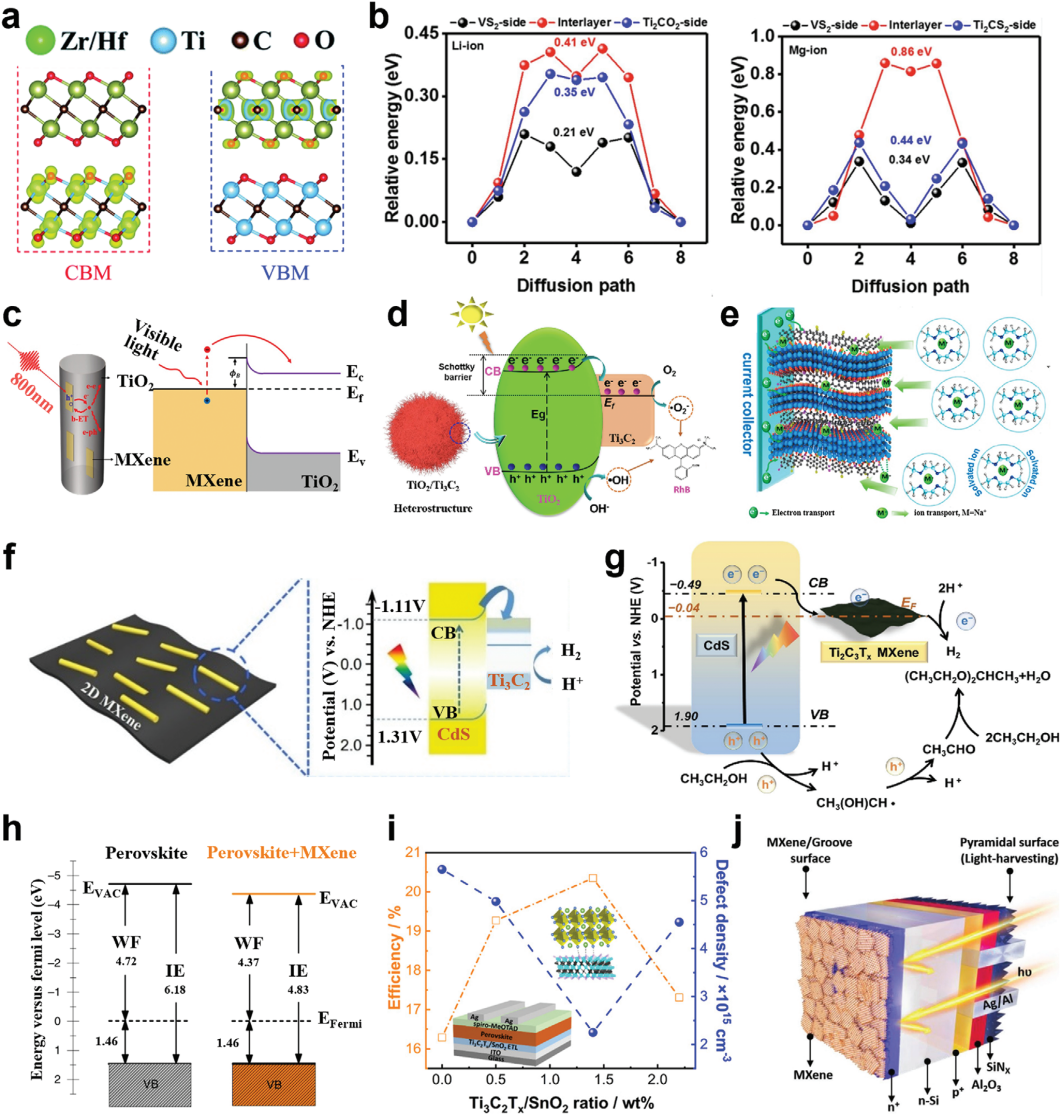
a) The CBM and VBM energy‐band‐decomposed resolved charge densities for the Ti_2_CO_2_/Zr_2_CO_2_ and Ti_2_CO_2_/Hf_2_CO_2_ heterostructures. Reproduced with permission.^[^ ^122^ ^]^ Copyright 2015, Royal Scoiety of Chemistry. b) Diffusion energy profiles for Li and Mg atom on VS_2_/Ti_2_CO_2_ and VS_2_/Ti_2_CS_2_. Reproduced with permission.^[^ ^206^ ^]^ Copyright 2021, Elsevier. c) Illustration of carrier kinetics diagram of TiO_2_/Ti_3_C_2_T*_x_* composite under photoexcitation and energy diagram of metal/semiconductor TiO_2_/Ti_3_C_2_T*_x_*. Reproduced with permission.^[^ ^209^ ^]^ Copyright 2021, American Chemical Society. d) Schematic of photocatalytic mechanism for TiO_2_/Ti_3_C_2_ heterostructure under light irradiation. Reproduced with permission.^[^ ^210^ ^]^ Copyright 2021, Elsevier. e) Na^+^ conduction kinetics of PAQS@MXene hybrid electrodes.^[^ ^211^ ^]^ cf) Illustration of 1D CdS/2D MXene Schottky heterojunction for hydrogen production. Reproduced with permission.^[^ ^212^ ^]^ Copyright 2020, Elsevier. g) Schematic diagram of reaction mechanism ethanol conversion on CdS–MX composite and photocatalytic H_2_ evolution under visible light irradiation in ethanol containing 30 mm H_2_SO_4_. Reproduced with permission.^[^ ^213^ ^]^ Copyright 2020, Elsevier. h) Energy scheme for undoped and MXene‐doped perovskite concerning the *E*_Fermi_. IE: ionization energy. *E*_VAC_, vacuum level. Reproduced with permission.^[^ ^214^ ^]^ Copyright 2021, Springer Nature. i) Schematic of the interactions between Ti_3_C_2_T*_x_* MXenes and perovskite. Reproduced with permission.^[^ ^216^ ^]^ Copyright 2021, American Chemical Society. j) Device architecture of Ti_3_C_2_T*_x_* MXene/n^+^np^+^‐Si solar cell. Reproduced with permission.^[^ ^5c^ ^]^ Copyright 2019, Wiley‐VCH.

Noteworthy, vdW heterojunction can also be formed in the MXenes of different combinations of elements. As shown in **Figure** **12**a, CBM is occupied by d‐electron of Ti atom and p‐orbit of O atom from Ti_2_CO_2_ layer, while VBM is contributed by p‐state of C atom and O atom from Zr_2_CO_2_ (Hf_2_CO_2_) layer. The indirect bandgaps featured in that band diagram of Ti_2_CO_2_/Zr_2_CO_2_ and Ti_2_CO_2_/Hf_2_CO_2_ heterojunction can delay the recombination rate of the photoinduced electrons and hole, and the slight energy difference between the direct bandgap and the indirect bandgap greatly reduces the influence on the optical absorption. This typical heterostructure facilitates the transmission of photoexcited electrons and holes in different layers, realizing the detection and conversion of photoenergy.^[^ ^122^ ^]^ Fan and co‐workers^[^ ^206^ ^]^ explored the electronic properties of the VS_2_/Ti_2_CT_2_ (T = O and S) structure, and it showed that the incorporation of VS_2_ improved the conductivity and the adsorption strength to Li^+^, Na^+^, Mg^2+^ of the heterostructure. VS_2_/TiCO_2_ and VS_2_/TiCS_2_ have relatively low diffusion energy barriers for Li and Mg ions, respectively, indicating that they are preferable anode materials for Li‐ion and Mg‐ion batteries (Figure 12b).

The application of MXene‐based heterojunction is very diversified. For the TiO_2_/MXene contact interface, the modulation of the interfacial electron transfer and adhesion will be helpful to modulate the heterostructure for gas sensitivity,^[^ ^207^ ^]^ energy storage applications.^[^ ^167^, ^208^ ^]^ For the plasma‐treated MXene/TiO_2_, even at SBH of 1.08 eV, the plasma barriers generated by the photoexcited MXenes were transferred to the CBM of TiO_2_ through the Schottky barrier at a fast time constant of 180 fs^[^ ^209^ ^]^ (Figure 12c). Noh and co‐workers^[^ ^210^ ^]^ developed a microscale safflower TiO_2_ heterostructure composed of nanorods. The photocatalytic mechanism is shown in Figure 12d. There are more complex interactions between organic molecules and MXenes, such as classical interactions, polymerization, strong polymerization, catalytic decomposition of organic molecules. More recently, Wang and co‐workers^[^ ^211^ ^]^ proposed that conjugated quinone and MXenes constructed a PAQS@Ti_3_C_2_T*_x_* MXene hybrid sandwich structure through an in situ polymerization‐assembly strategy (PAQS: polyanthraquinone sulfide), which relied on the fast carrier transport ability of MXenes, and provided the possibility to realize efficient organic sodium‐ion batteries (Figure 12e). The cadmium sulfide (CdS) nanorods can be generated by in situ assembly solvothermal method on Ti_3_C_2_ nanosheets. The electrons formed by the Schottky junction at the heterogeneous interface of CdS/MXenes effectively promoted the separation and transfer of electron–hole pairs^[^ ^212^ ^]^ (Figure 12f). In addition, MXenes can enhance the separation and migration of photogenerated carriers in CdS, optimize the pore structure and specific surface area of the composite, and thus realize the efficient photocatalytic coupling redox reaction^[^ ^213^ ^]^ (Figure 12g). Frequently, the fabrication of high‐performance perovskite solar cells (PSCs) often combines perovskite with a work function adjustable material. As shown in Figure 12h, the work function of perovskite decreases from 4.72 to 4.37 eV after MXene is inserted into the mTiO_2_ (mesoporous) and perovskite layers, reducing the charge recombination rate at the perovskite interface.^[^ ^214^ ^]^ The planar PSCs were integrated by spin‐coating method, which exhibited that the maximum power conversion efficiency (PCE) could reach 17.17%.^[^ ^215^ ^]^ Furthermore, the hybrid film composed of SnO_2_ nanoparticles/Ti_3_C_2_T*_x_* MXene nanoflakes is used as a charge transport layer for PSC. When the ratio of Ti_3_C_2_T*_x_*/SnO_2_ is changed, the defect density of the polycrystalline perovskite film is remarkably reduced from 5.65 × 10^15^ to 2.25 × 10^15^ cm^3^, as shown in Figure 12i.^[^ ^216^ ^]^ Analogous to the graphene and n‐Si heterojunction, He and co‐workers^[^ ^5^ ^]^ used the drop casting method to fabricate the Ti_3_C_2_ MXene–n‐Si^+^‐contacted solar cell, as shown in Figure 12j, showing the maximum conversion efficiency of the final solar cell up to 11.5%. These electronic devices with MXene heterojunction exhibit superior performance compared with the traditional Au/Ti contact device in terms of responsivity, quantum efficiency, and dynamic range. As the manufacturing process only included simple photolithography, spin‐coating, and liftoff, the device miniaturization of photonic integrated circuits could be achieved.^[^ ^217^ ^]^

## 4 MXene Contacts for Printable Electronics

Printed electronics are defined as these electronic devices and circuits prepared by printing technology, including printed transistors, printed memory devices, printed batteries, printed conductive films, printed antennas, and other devices related to electronic circuits.^[^ ^218^ ^]^ Thanks to good chemical and mechanical stability, ideal printability, simple, fast, low‐cost surfactant‐free solution process, MXene inks with different electronic properties have been used to manufacture various multifunctional electronic devices with printing processes. The printing method can make the devices larger area, more flexible, lower cost. Based on the unique structure of MXenes, abundant controllable surface groups, and a wide range of work function, various MXene‐based heterojunction contact types can be available for integrated electronic applications. This section summarizes the latest technologies and application scenarios for manufacturing MXene‐based printed electronic devices.

### 4.1 Electronics and Components

#### 4.1.1 Energy Storage Devices

The MXene‐based device by printing method is most widely applied as supercapacitor. The porous structure in MXene‐based nanomaterials with printed structures effectively facilitates electrolyte permeation and ion transmission, which makes MXene‐based capacitors have favorable energy density and mechanical properties.^[^ ^219^ ^]^ For one thing, the capacitance is determined by the inherent characteristics of the prepared material. For another, the operating voltage can be amplified by matching the asymmetric supercapacitor with different voltage windows.^[^ ^220^ ^]^

**Figure 13:**
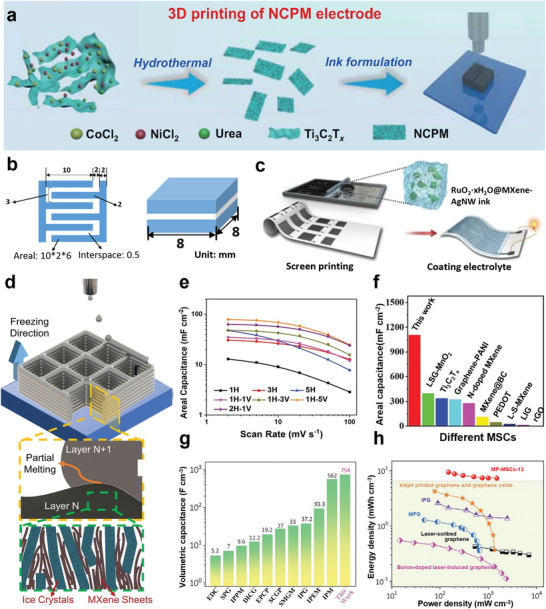
a) Illustration of the NCPM synthesis and the 3D printing process. Reproduced with permission.^[^ ^76^ ^]^ Copyright 2020, Springer Nature. b) The effective area of MSC printed by screen printing (left) and extrusion(right).^[^ ^69^ ^]^ Copyright 2019, Wiley‐VCH. c) The fabrication processes for screen‐printed flexible MSC devices. Reproduced with permission.^[^ ^9^ ^c]^ Copyright 2019, Wiley‐VCH. d) Schematic diagram of the 3DFP process used for the prepare the 3D Ti_3_C_2_T*_x_* aerogel. e) The areal capacitance of different 3DFP MSC devices. The fabricated devices would be labeled MSC‐*X*H‐*Y*V, where *X* and *Y* represent the number of printed layers aligned on horizontal (H, air dried) and vertical (V, freeze printed/dried) sheets, respectively. Reproduced with permission.^[^ ^78^ ^]^ Copyright 2020, Wiley‐VCH. f) Compare with the areal capacitance of 3DFP MSC to those of other reported MSCs. Reproduced with permission.^[^ ^18^ ^]^ Copyright 2021, Wiley‐VCH. g) Volume capacitance of MP‐MSC‐12 tested at 15 µA cm^−2^ compared to previously reported MSCs. EDC: electrophoretic deposition carbon, SPG: self‐aligned printed graphene; IPPM: inkjet‐printed PEDOT/MnO_2_; DLSG: Digital versatile disc (DVD) laser‐scribed graphene; EPGP: extrusion‐printed graphene oxide (GO)/polyaniline (PANI)/PEDOT; SCGP: spray‐coated graphene/PEDOT; SMGM: spray‐masked graphene/MXene; IPEG: inkjet‐printed electrochemically exfoliated graphene; IPM: inkjet‐printed MXenes. h) Ragone plot of MP‐MSC‐12 compared with previously reported MSCs. Reproduced with permission.^[^ ^21^ ^]^ Copyright 2021, Wiley‐VCH.

The most common functionalization ways are in situ growth, chemical modification, and adsorption doping. In situ production of NiCoP nanowires on the surface of nanosheets produces NiCoP/MXenes (NCPM) structure. 3D‐printed NCPM and activated carbon are used as positive and negative electrodes of MSC, respectively. Printing electrode with hierarchical pores and facile charge transport endows capacitors with higher rate capability and cycle stability^[^ ^76^ ^]^ (**Figure** **13**a). N‐doped chemically modified MXene nanosheets were prepared by the melamine–formaldehyde template method to improve the conductivity and redox activity. The area capacitance of N‐doped MSC is significantly higher than that of pure MXenes^[^ ^69^ ^]^ (Figure 13b). Besides, the hydrous RuO_2_ nanoparticles are anchored on the Ti_3_C_2_T*_x_* MXene nanosheet, which inhibits the self‐stacking phenomenon. The obtained RuO_2_@MXene nanosheets and AgNWs are prepared into printing ink (Figure 13c). The introduction of nanoparticles enlarges the interlayer spacing of MXenes and promotes the transfer of electrons from MXenes to RuO_2_. The fabricated MSC exhibited a volume capacitance of 864.2 F cm^−3^ at a scan rate of 1 mV s^−1^.^[^ ^9c^ ^]^

In addition, the vertical alignment of the MXene sheets can also reduce sheet stacking, bringing the ions closer to the electrodes for maximum capacity electrochemical cycling.^[^ ^100^ ^]^ In extrusion‐based multilayer 3D printing, defective voids occur between adjacent layers. This is due to insufficient adhesion of the deposited layer interface induced by intermolecular diffusion. In the 3DFP method, the *N*+1 layer deposited at room temperature results in melting of the frozen upper surface of the *N* layer. The low viscosity ink fills the voids between the layers utilizing surface tension and gravity, and melts *N* layer with *N*+1 layer, thus reducing the formation of voids or boundary between deposited layers (Figure 13d). Finally, the unidirectional frozen ink forms a close‐packed network structure between the ice crystals, and the MXene aerogel is obtained after freeze drying. Accounting for the microstructure anisotropy of the prepared interdigitated Ti_3_C_2_T*_x_* electrodes, the conductivity also exhibited anisotropy. Moreover, changing the orientation of MXene sheets and designing the electrode structure also has a significant effect on the area capacitance (Figure 13e).^[^ ^78^ ^]^

In order to make full use of the reaction “trash,” a precipitate consisting of an unetched MAX phase and an unlayered MXene (m‐MXene) after stratification is collected, leaving a few percent of the layered nanosheets (d‐MXene) in the precipitate. On the one hand, d‐MXene is cross‐combined with the layered particles to adjust the rheological characteristics. On the other hand, d‐MXenes can act as a conductive adhesive bridging the particles, wrapping the MAX particles to form a continuous metal network, maintaining a smooth printed circuit and good mechanical stability. Furthermore, m‐MXenes and MAX fillers with higher capacitance than d‐MXenes enable improvement of proton intercalation kinetics, and enhance the charge storage properties of the deposited ink MSC. Screen printing allows for efficient printing of each conductive track and integrated circuit pattern without the use of an additional current collector or polymer binder during the entire process. The area capacitance of MSC prepared in combination with the sulfuric acid–polyvinyl alcohol (PVA) gel electrolyte was 158 MF cm^−2^, maintaining 98.8% of the initial capacitance after 17 000 cycles.^[^ ^66^ ^]^

More recently, it was found that a flat substrate with strong adsorption and wettability to the ink facilitates ink adhesion, which was used in the fabrication of micro‐supercapacitors. The MXene‐based micro‐supercapacitors printed with ten printed layers (MX‐MSCs‐10L) constructed by screen printing show area capacitance of 1108 mF cm^−2^ (Figure 13f). MX‐MSCs‐5L does not show any attenuation after 10 000 cycles.^[^ ^18^ ^]^ In addition, PVA and MXene ink are mixed with a cross‐linking agent borax to form MXene‐based hydrogel to manufacture a pressure sensor. Although the self‐discharge rate of MSC was degraded by the ability to customize and adjust the chemical interface, the self‐discharge rate reached 1.2–0.7 V h^−1^. After reconstruction, the self‐discharge time was extended from 2.3 to 1.8 V over 9 h. It was also reported that fully flexible self‐powered integrated system could be constructed based on the MXene device above, including a series‐connected silicon solar cell as the energy collector, MXene‐based lithium‐ion microbatteries for energy storage, hydrogel pressure sensor for signal detection to sensitively detect the bending of body parts with high response of 35 ms.^[^ ^18^ ^]^ Likewise, a highly conductive PH1000 mixed with MXenes was employed to form MP inks, by which the device was prepared to show a volume capacitance of up to 754 F cm^−3^, and energy density of 9.4 mWh cm^−3^ at a power density of 150 mWh cm^−3^ (Figure 13g,h). When the silicon solar cell was connected in series with the printed MSC and the temperature sensor (TS) to construct a self‐powered integrated system, the highest response value of the self‐powered TS reached about 2.0% at 50 °C.^[^ ^21a^ ^]^

#### 4.1.2 Sensors and Actuators

**Figure 14:**
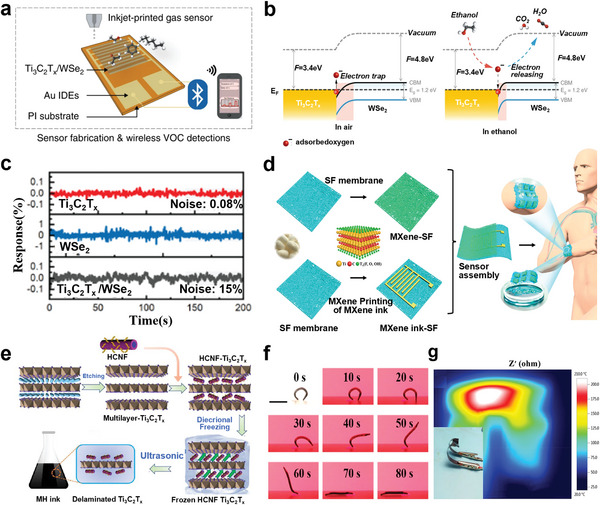
a) Illustration of inkjet‐printed gas sensors using wireless monitoring system to detect volatile organic compounds. b) Energy band diagram of Ti_3_C_2_T*_x_*/WSe_2_ in air (left) and ethanol (right) showing the variation of depletion layer on the interaction between adsorbed oxygen species and ethanol molecules. c) Electrical noise measurements of the pristine Ti_3_C_2_T*_x_*, WSe_2_, Ti_3_C_2_T*_x_*/WSe_2_ electrical noise measurements. Reproduced with permission.^[^ ^73^ ^]^ Copyright 2020, Springer Nature. d) Schematic diagram of the fabrication procedure of the breathable and degradable MXene/protein‐nanocomposite‐based pressure sensor. Reproduced with permission.^[^ ^221^ ^]^ Copyright 2021, American Chemical Society. e) Schematic diagram of the fabrication of MH inks via assisted intercalation and the freeze–thaw method. Reproduced with permission.^[^ ^9^ ^b]^ Copyright 2021, American Chemical Society. f) An optical image of an induced shape transition of a binary‐layered MXene hydrogel (BLMH) actuator fixed at one end to a reference. Scale: 2 cm. Reproduced with permission.^[^ ^222^ ^]^ Copyright 2021, American Chemical Society. g) Infrared thermography of MXene/epoxy actuator. Inset is the bending state diagram of the actuator. Reproduced with permission.^[^ ^13^ ^]^ Copyright 2021, American Chemical Society.

MXene‐based functional materials have been widely explored as electrodes or active layers in the sensors with responses to gas, moisture, pH, pressure, and light. The sensors were found to effectively transform the determining information into the external stimuli following a specific rule. For example, the abundant surface groups of MXenes furnish a large number of gas adsorption sites, so intrinsic MXenes have excellent gas sensing properties.^[^ ^207^ ^]^ Based on the discussion of the two dimensional material and MXene heterojunction in the previous section, Ti_3_C_2_T*_x_*/WSe_2_ contact system has desirable charge transfer characteristics. By inkjet printing, the integrated flexible wireless sensor system was fabricated, as shown in **Figure** **14**a. As the energy band diagram of the hybrid describes (Figure 14b), the electron movement direction tends to flow from Ti_3_C_2_T*_x_* to WSe_2_ in the Ti_3_C_2_T*_x_*/WSe_2_ contact system. The hybridization of the system enables to reduce the sheet resistance by more than four orders of magnitude, and exhibits the highest gas response and low‐electrical noise^[^ ^73^ ^]^ (Figure 14c). Pressure sensors are extensively concerned in wearable artificial skin, medical detection, artificial intelligence, and other fields. As reported, the fabrication of MXene films with anisotropic alignment and closely packed deposition morphologies was achieved by solvent evaporation inducing alignment of MXene nanoparticles to show a high response characteristic at low pressure, resulting in an increased film resistance. When the applied pressure exceeds 20 kPa, the microstructure deformation reaches saturation and the sensitivity decreases.^[^ ^9^ ^]^

The sensor device with high degradability has been highly expected in recent years. Degradable materials, like silk fibroin nanofiber membrane, were already used as the substrate for depositing the MXene electrode with screen printing (MXene–SF ink). The MXene–SF inks were assembled into piezoresistive sensors through supramolecular interaction (Figure 14d), indicating high sensitivity up to 298.4 kPa^−1^ and a wide working range of 39.28 kPa. Moreover, the sensor exhibits rapid autocatalytic hydrolysis and bulk degradation after 9 days in alkaline solution.^[^ ^221^ ^]^ “Holocellulose” is the total polysaccharide fraction of native wood or straw, which has high biodegradability and biocompatibility. Holocellulose nanofibers with the “core–shell” structure were inserted into the MXene nanosheets, MH ink was obtained upon several cycles of directional freezing and thawing (Figure 14e). Through the intervention on the interlayer spacing of MXenes and the surface functional groups, the sensor device prepared by MH ink has a uniform and stable response curve for stress and strain, temperature and humidity, which exhibits the applicability in multifunctional sensing.^[^ ^207^ ^]^

The photothermal effect of MXenes has also been investigated and applied to sensitive actuators. Utilizing thermal response anisotropy of the hydrogels, a binary‐layered MXene hydrogel actuator was constructed. As a result, the obtained actuator can be overturned 180° and flattened after 80 s near‐infrared exposure^[^ ^222^ ^]^ (Figure 14f). Similarly, MXene/epoxy resin composite film was prepared by compounding diluted highly cohesive dough‐like MXenes with the epoxy resin film. The composite membrane can also exhibit extraordinary flexibility under low‐pressure driving. The diluted MXene slurry can also be used as a material for 3D printing or DIW ink patterning^[^ ^13^ ^]^ (Figure 14g).

#### 4.1.3 Transistor and Logic Circuit

Transistors have a wide range of digital and analog functions, including amplification,^[^ ^147^, ^223^ ^]^ switching,^[^ ^224^ ^]^ voltage regulation,^[^ ^225^ ^]^ oscillation,^[^ ^226^ ^]^ mixing and frequency conversion.^[^ ^144^ ^]^ It is the core of modern communication and display technology electronic components and is deemed one of the landmark inventions in modern history.^[^ ^156a^ ^]^ As discussed in the contact section, the formation of Ohmic contact between the electrode and the semiconductor is particularly essential, and the high potential barrier increases the difficulty in charge transport and degrades the performance of device. Moreover, the pinning effect caused by the surface state still makes the regulation of the interface potential barrier a challenge.

**Figure 15:**
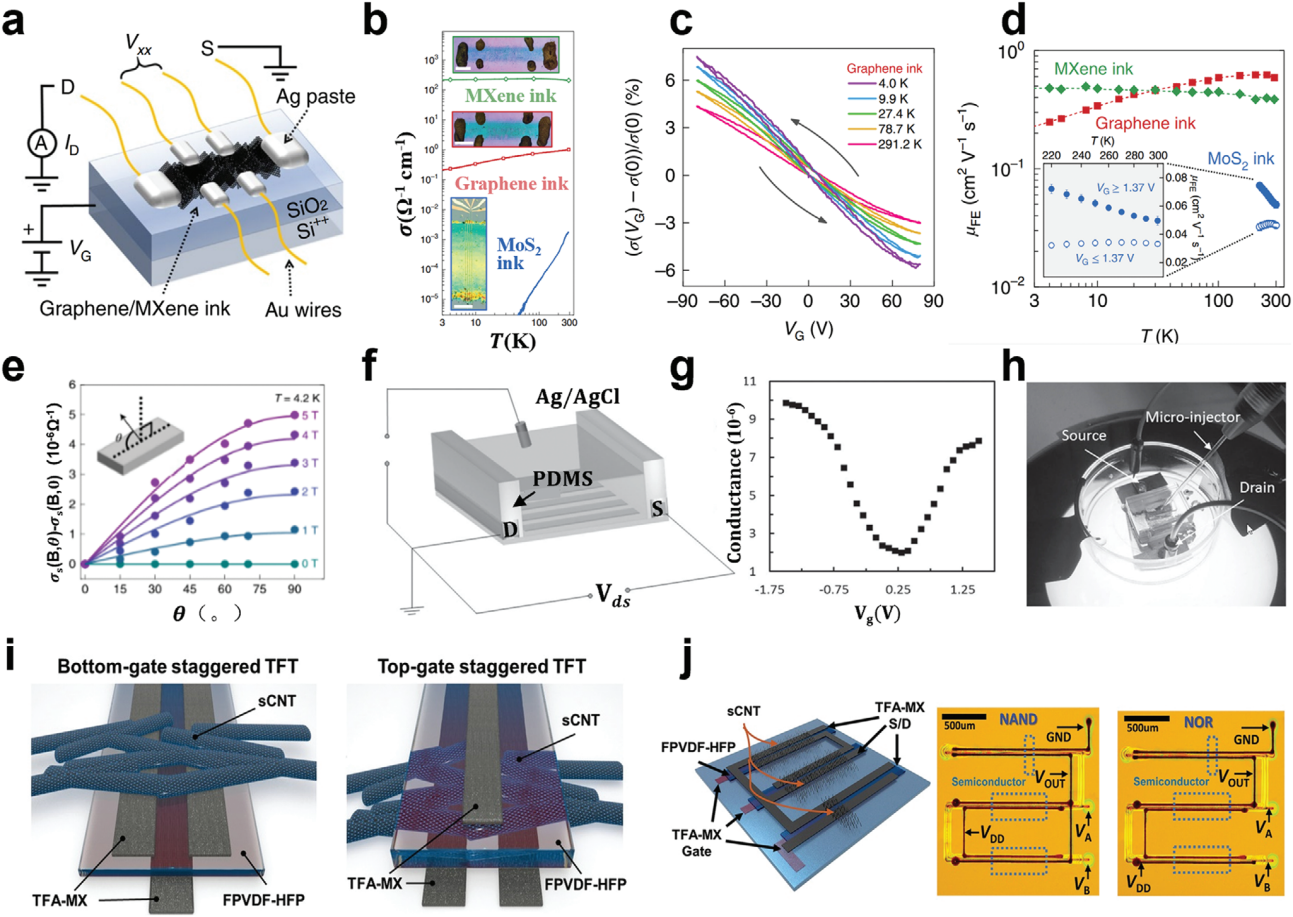
a) Schematic of inkjet‐printed graphene/MXene field‐effect device. S, D: source, drain. b) At gate voltage *V*_G_ = 0, the conductivity *σ* expressed in logarithmic scale is a function of the field effect transistor temperature *T*. The solid blue, solid red, and solid green lines are the *σ* values for the printed MoS_2_‐ink device, the printed graphene‐ink device, and the printed MXene‐ink device, respectively. The inset represents the printed optical image (green border), printed graphene‐ink device (red border), and printed MoS_2_‐ink device (blue border). c) Conductivity ratio [*σ*(*V*_G_) − *σ*(0)]/*σ*(0) as a function of *V*_G_ for increasing *T* in the printed MXene‐ink device. d) The mobility *µ* of a field‐effect transistor (FET) device is a logarithmic function of *T*. Printed graphene‐ink devices (red squares), printed MXene‐ink devices (green diamonds), and printed MoS_2_‐ink devices (hollow blue circles, *V*_G_ ≤ 1.37 V, filled with blue circles, *V*_G_ ≥ 1.37 V). Inset shows a magnified view of MoS_2_ mobility over a linear range.^[^ ^10a^ ^]^ e) For different values of *B*, the anisotropic permeability is a function of the angle *θ* between *B* and the substrate plane. Solid circles are experimental data at *T* = 4.2 K. The solid line fits the sin(*θ*) correlation. Reproduced with permission.^[^ ^10a^ ^]^ Copyright 2021, Springer Nature. f) The photograph shows the MXene–FET device connected to microinjector used to precisely control the pulsed release of dopamine in the biosensor chamber. g) Record the variation in conductivity of the MXene device with 100, 250, 500, 1000 × 10^9^ m dopamine. h) Schematic of biosensor device based on MXene field‐effect transistor. Reproduced with permission.^[^ ^83^ ^]^ Copyright 2016, Wiley‐VCH. i) Schematic illustrations of the bottom‐gate staggered TFT (left) and top‐gate staggered TFT (right) prepared with EHD‐printed TFA–MX electrodes, sCNT active layer, and FPVDF‐HFP dielectric layers. j) Schematic illustration (left) and top‐view optical microscope (OM) images (right) of all‐printed logic gates (NAND and NOR) integrated by bottom‐gate staggered TFTs. Reproduced with permission.^[^ ^10^ ^]^ Copyright 2021, Wiley‐VCH.

All printed transistor devices require all materials used for manufacturing to be dissolved to obtain stable ink with suitable printability.^[^ ^227^ ^]^ MXene has excellent solution properties without any additives, thus making it an ideal printed transistor material. A printed transistor has been constructed based on inkjet‐printed MXene active layer^[^ ^10^ ^]^ (**Figure** **15**a). Compared with graphene and MoS_2_ ink, the conductivity of MXenes is almost temperature‐independent. This suggests that charge transport occurs in the extended state (Figure 15b). The conductivity of MXenes increases with the increase of *V*_G_ without any hysteresis, indicating that MXene has outstanding intrinsic electron conduction property (Figure 15c). Within the same temperature increase range, the mobility of MXene‐based transistors slightly increases from 0.4 to 0.5 cm^2^ v^−1^ s^−1^ compared with other 2D materials. Similar to disordered metal systems, the charge carrier scattering during transport is basically due to the internal defects^[^ ^10^, ^228^ ^]^ (Figure 15d). It has been found that the surface conductivity *σ*_s_ is a linear relationship as a function of log(*T*), indicating that charge transport is similar to granular metals. The anisotropic permeability curve follows the expected sinusoidal function correlation^[^ ^10^ ^]^ (Figure 15e). In the transfer printed field effect transistors, Polydimethylsiloxane PDMS is applied to separate the electrode from the bath solution to improve the leakage current. The transferred micropattern electrical path can be controlled by the gate voltage, and a V‐shaped ambipolar field‐effect characteristic similar to that of graphene is observed (Figure 15f,g). Based on this, a high‐sensitivity biological detection device was fabricated, composed of a transistor circuit for electrical measurement and a microinjector^[^ ^83^ ^]^ (Figure 15h).

Kim and co‐workers^[^ ^10b^ ^]^ proposed that an all‐printed thin‐film transistor (TFT) is constructed by using fluorophenyl azide‐3F‐based cross‐linked poly(vinylidene fluoro‐*co*‐hexafluoropropylene) (namely, FPVDF‐HFP) dielectrics and printed carbon nanotube semiconductors (sCNT) (Figure 15i). The p‐type transport favorable for hole injection is formed at the MXene/sCNT interface. By assembling two or three devices to construct a logic gate circuit, inverters exhibit typical static circuit characteristics. The diode‐loaded inverter can achieve a maximum voltage gain of 20 times at a high input current of close to 0 V. The complex logic gates are integrated by constructing TFTs (Figure 15j).

### 4.2 Organic Photovoltaic and Light‐Emitting Diodes

**Figure 16:**
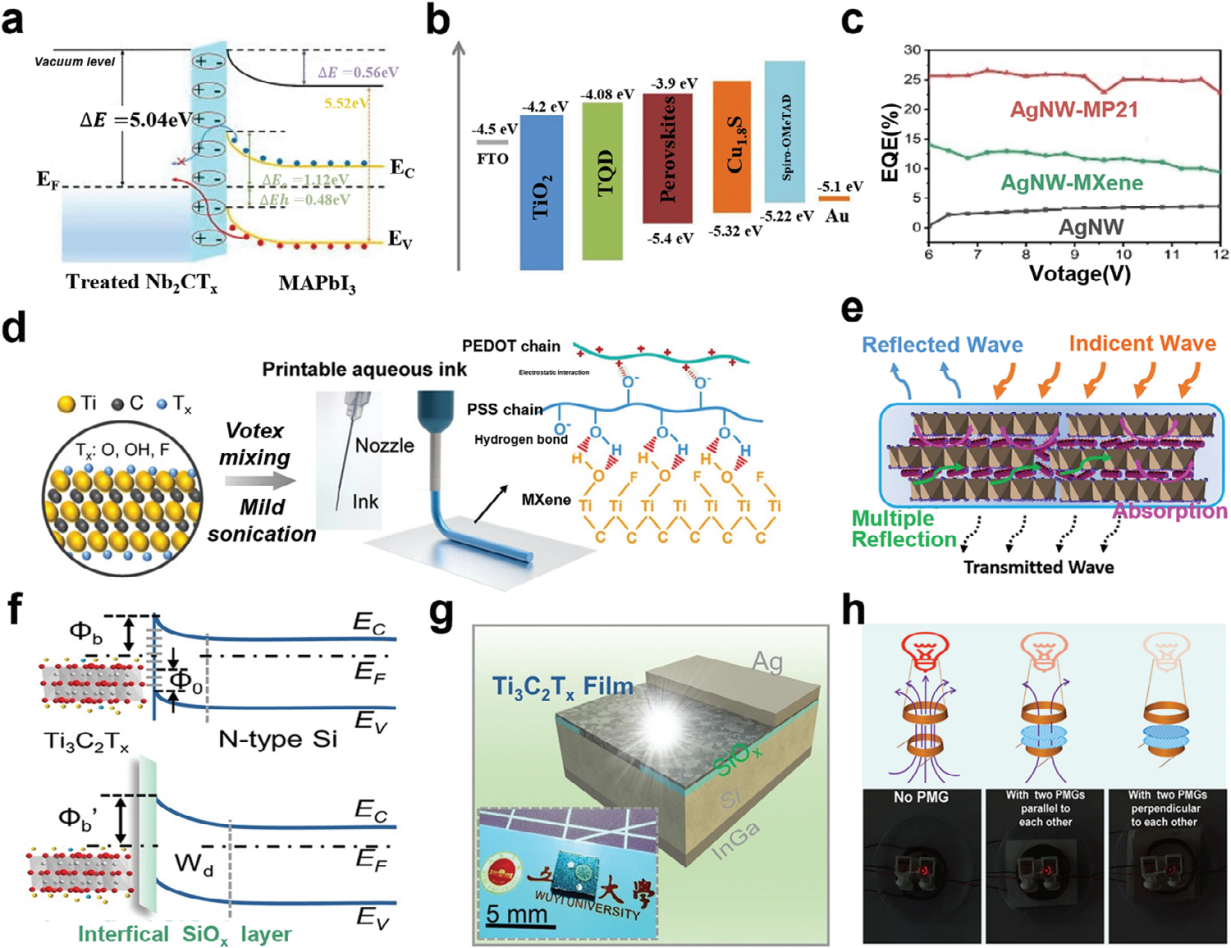
a) Energy level diagram of oxygen‐plasma‐treated Nb_2_CT*_x_*/MAPbI_3_. Reproduced with permission.^[^ ^15^ ^]^ Copyright 20202, Wiley‐VCH. b) Energy diagram of each layer of SnO2:F(FTO)/TiO_2_/TQD/perovskites/Cu_1.8_S/2,2',7,7'‐tetrakis(N,N‐di‐p‐methoxyphenylamine)‐9,9'‐spiro‐bifluorene (Spiro‐OMeTAD)/Au PSCs. Reproduced with permission.^[^ ^231^ ^]^ Copyright 2020, Wiley‐VCH. c) External quantum efficiency (EQE) of OLED devices using AgNW, AgNW–MXenes, and AgNW–MP21 (MXenes and PEDOT:PSS of mass ratio of 2:1) hybrid flexible transparent electrodes (FTEs). Reproduced with permission.^[^ ^232^ ^]^ Copyright 2020, Elsevier. d) Schematic of MXene‐functionalized PEDOT:PSS ink for extrusion printing. Reproduced with permission.^[^ ^16^ ^]^ Copyright 2021, Wiley‐VCH. e) Schematic of incident wave propagation through a printed product. Reproduced with permission.^[^ ^9^ ^b]^ Copyright 2021, American Chemical Society. f) Energy band diagram of a Ti_3_C_2_/Si heterojunction before and after interfacial modification, where *Φ*_0_ is the surface potential, *W*_d_ is the depletion width. g) Schematic illustration of the Ti_3_C_2_/SiO*_x_*/Si photodetector (PD), the inset is an actual device photograph. Reproduced with permission.^[^ ^64^ ^]^ Copyright 2021, Wiley‐VCH. h) Polarization and switching effects of the microgratings to electromagnetic waves in a wireless generator device. Reproduced with permission.^[^ ^23^ ^]^ Copyright 2022, American Chemical Society.

Along with a large number of studies on renewable photovoltaic energy, photoelectric conversion efficiency of solar cells (SCs) has been elevated tenfold in just one decade.^[^ ^229^ ^]^ In particular, the tunable work function of MXenes endows it a distinctive advantage in multi‐interface devices. The low temperature solution processability and printability of MXenes also make it compatible with photovoltaic manufacturing technology. Attributed to the high transparency, high conductivity, and carrier mobility of the MXenes, Ti_3_C_2_T*_x_* MXene is one of the commonly used materials for perovskite solar cells (PVSCs) and organic solar cells.^[^ ^230^ ^]^ As the charge transport layer of SC, the oxygen‐plasma‐modified MXene brings about uniformly increasing Ti—O bonds, and the interfacial charge transport efficiency is optimized by the modulation of work function and the reduction of interfacial trap states. Suitable wettability and surface‐free energy facilitate device stability.^[^ ^15^, ^110^ ^]^ Likewise, the oxygen functional group acts on the work function of Nb_2_CT*_x_* by increasing the surface dipole, and the energy band of the perovskite/MXene interface is bent, which is beneficial to diminishing the charge accumulation and energy loss at the interface. At the same time, the built‐in potential and open‐circuit voltage of PVSCs are well modulated^[^ ^15b^ ^]^ (**Figure** **16**a). It is reported that the hybrid of MXenes and metallic SWCNTs (MXene/m‐SWCNTs) could significantly enhance the charge extraction at the interface of SnO_2_/perovskite. The work function of MXene/m‐SWCNTs mixture was about 4.3 eV, which completely matched the work function of SnO_2_/perovskite of 3.2 eV. The manufactured PSC achieved more than 21% PCE, and its fill factor was close to 0.80.^[^ ^15a^ ^]^ In addition, 2D MXene quantum dots (TQDs) are often used as the interlayer and buffer layer of SC. It can be used to enlarge the crystal size and conductivity of perovskite and develop the transmission gradient of TiO_2_ and perovskite and enhance the charge transmission efficiency^[^ ^231^ ^]^ (Figure 16b).

Light‐emitting diodes are promising in the field of high‐resolution screens and lighting. Flexible light‐emitting diodes require the development of solution‐processable transparent electrodes in order to achieve high efficiency with low power consumption and high brightness, and to be combined with a variety of processes. However, the poor interface contact between MXene thin film and the carrier transport layer is the main issue for the undesirable performance of the device. Vacuum annealing and chemically neutralized polymeric hole injection layer (HIL) can not only protect MXenes from oxidation, but also form a gradient work function inside the HIL achieving effective hole injection.^[^ ^5^ ^]^ When in contact with the silver nanowires as a dopant, the doping of MXenes lowers the surface roughness. The evaporation of the solvent further enhances the electrical contact between the nanowires and the nanosheets and promotes the adhesion of the substrate. With the addition of the Poly(3,4‐ethylenedioxythiophene/poly(styrenesulfonate) (PEDOT:PSS), the binding energy of the chain was reduced by the electrostatic interaction between nanosheets and chains. After that, it was converted into a quinone structure, which promoted charge redistribution and increased the charge delocalization and conductivity. The maximum external quantum efficiency of fabricated flexible light‐emitting diode reaches 25.9% (Figure 16c), and the excellent electroluminescent performance is attributed to the effect of the dopant on the surface state of AgNWs, resulting in increasing work function of 0.2 eV, availably reducing the interface barrier and facilitating hole injection.^[^ ^232^ ^]^

### 4.3 Electromagnetic Interference Shielding

The proliferation of mobile wearable devices with various wireless technologies increases the frequency and duration of human exposure to electromagnetic fields, which causes interference and safety problems, so it is necessary to select appropriate materials for electromagnetic (EM) protection. MXenes take on lightweight and strong mechanical deformability compared to metals. In the internet of things field, MXenes enable wireless communication between wearable and mobile devices, the stretchable antenna can facilitate mechanically stable wireless transmission while attenuating EM absorption by the body.^[^ ^84^, ^233^ ^]^ EMI shielding cuts off the propagation path of electromagnetic waves employing free carrier absorption, interface polarization, dipole polarization, and magnetic absorption. Using absorption and reflection enables to form a closed environment without disturbing the external magnetic field.^[^ ^234^ ^]^ Therefore, the better the conductivity and magnetic conductivity of the material, the higher the shielding effectiveness. Functionalized MXene inks can be obtained by compounding with PEDOT:PSS.^[^ ^16^, ^79^ ^]^ A highly conductive and robust hydrogel was obtained using extrusion 3D printing in combination with freeze–thawing (Figure 16d). In a high intensity alternating electromagnetic field, the freely moveable carriers in that printing path produce a scattered or induced field in response to the incident wave. In particular, when the printed line distance is less than the incident wave length, the conductive path easily interacts with the incident wave to degrade the electromagnetic field strength.^[^ ^22^, ^79^ ^]^ The porous structure in the highly conductive hydrogel absorbs water molecules in the environment to customize EMI shielding performance.^[^ ^16b^ ^]^

Similarly, sulfated‐HCNF‐intercalated MXene ink was screen‐printed into circular patterns on a cellulosic paper substrate.^[^ ^9b^ ^]^ The distinctive “core–shell” structure of HCNF forms a conductive network with MXenes. When the electromagnetic wave is brought into contact with the nanosheet with high surface charge density, the carriers on the nanosheet will absorb the energy of the electromagnetic microwave and transmit it along the conductive network. Relaxation losses convert electromagnetic energy into thermal energy. An additional small portion of the incident electromagnetic microwave is reflected at the interface of the air and the print. Moreover, the layered structure of the nanosheet layer captures and weakens the electromagnetic microwave through repeated reflection, absorption, and scattering. Meanwhile, the “core–shell” structure induces interface polarization and improves the electromagnetic microwave absorption capacity, and the print finally presents excellent shielding characteristics after absorbing high microwave and reflecting low microwave^[^ ^9b^ ^]^ (Figure 16e).

### 4.4 Others

Interface engineering is an available means to ameliorate the photoelectric characteristics of photodetector (PD).^[^ ^64^, ^235^ ^]^ Hanging bonds on the Si surface cause severe Fermi pinning effect, give rise to the barrier height being affected by the surface state. The energy band diagram manifests the formation of Schottky barrier and depletion region when MXene contacts with n‐Si. By introducing a passivated dielectric layer, a barrier layer is formed at the heterojunction interface to inhibit dark leakage current, the responsiveness and detection rate of PD are improved (Figure 16f,g). Under illumination, the built‐in electric field separates the photogenerated electron–hole pairs in Si, as well as tunneling through the interface barrier pulls the holes toward MXenes, where the electrons move toward Si and are collected by the back electrode.^[^ ^64^ ^]^

Printed electronics and silicon‐based electronics are not a substitute for each other, but complement each other. Combining the benefits of MXenes and silicon‐on‐insulator, inkjet printing of MXenes onto a silicon microring resonator (MRR) was used. MXene absorbs and heats MRR by its strong photothermal effect. The resonance light in MRR significantly enhances the interaction between light and matter. All‐optical modulator satisfying all‐optical phase modulation and intensity modulation, not only can realize large‐scale integration interconnection, but also has great advantages in compatibility with the complementary metal‐oxide‐semiconductor (CMOS) process.^[^ ^144^ ^]^ Zhang and co‐workers^[^ ^23^ ^]^ grafted the prepolymerized poly(dopamine) macromolecules onto the surface of MXene, and significantly optimized the rheological properties of the ink to adapt to a variety of high viscosity processing technologies. Based on the polarization and switching effects of the microgratings to electromagnetic waves (Figure 16h), the printed MXene micropattern acts as a switch that prevents or allows the transmission of electromagnetic waves, and the resulting polarizer has a freely switchable and quantitatively controlled microwave transmission of 93.6–2.4%.

## 5 Conclusion and Future Perspectives

This review comprehensively summarized the background, operation principle, physical mechanism of MXene contacts in the printed electronics. From the viewpoint of chemical synthesis to physical deposition, MXenes can be used as a versatile platform for fabricating conductive electrodes with tunable work functions in a wide range (4.4–5.8 eV), thus enabling to effectively reducing contact barriers. This character has not been yet observed in other conductors. In addition, MXene and its derivatives could be well dispersed in liquids to form colloidal inks, thereby perfectly satisfying the requirements of printed electronics. Moreover, the typical structure of the MXene nanosheets and their excellent contact controllability allow for different electronic device requirements, thus furnishing the possibility of integrating high‐performance printed electronic systems. Although there is a gap between the performance of MXene‐based printed electronic devices and silicon‐based microelectronic devices, the peculiarities of MXene‐based printed electronic products have attracted its exclusive market, such as transparent conductive films for photovoltaic device construction, flexible wearable devices, electronic integrated systems on flexible substrates, etc. The indicators that measure the progress of printed electronics include the minimum print size, carrier mobility, and the complexity and synergy of printed electronic systems. Therefore, new or optimized printing processes need to be explored to accommodate more electronic devices per unit area. The ideal energy band structure and contact barrier can be finally realized through accurate modulation of surface end groups, surface defects, work function, and Fermi pinning effect.

In the near future, MXene‐based printed electronics will continue to extensively attract attention from scholars especially in the fields of organic photovoltaics, organic integrated circuits, organic light‐emitting diodes, and organic heterojunction devices. In order to achieve printing of flexible integrated system with larger area, high performance, and high reliability, the challenging issues existing in the investigation on printing process and contact engineering of MXenes need to be addressed.

1. Ink formulations designed according to objective requirements—affected by the synthesis conditions, the surface groups of MXenes greatly depend on the synthesis conditions, and the preparation methods dominated by the HF etching and in situ etching can no longer meet the application requirements. Emerging synthetic methods, printing methods, and interaction strategies should be developed to explore green and safe controllable synthetic MXene‐based printing electronic materials.
2. Limitations of material types—numerous MXenes are existing only in theoretical studies, of which a few MXenes have been synthesized. The properties of many predictions have not been verified by experiments. In particular, the influence of different MXene element combinations on the device of performance should be deeply investigated.
3. Limitations of the printing process—printing is a tool with both advantage and disadvantage. The minimum circuit feature size of modern integrated circuits is less than 20 nm. The high‐resolution printing technology, like inkjet or gravure printers can only yield a few micrometer patterns, far less than that of photolithography. Besides, in multilayer stack printing, ensuring preferable alignment accuracy and upgrading the printed surface morphology are also essential scientific issues. In addition, the rheological properties of MXenes following surface modification should be further studied to improve the quality of the ink, and to determine the appropriate processing route, achieving the parameter regulation of the ink in multiple dimensions.
4. Deep understanding of contact mechanism—gaining insight into the relationship between interface contacts and device performance, further mechanisms and explanations of materials, structures, and properties in contact with MXenes remain to be explored. The mechanism of MXene surface modification on bandgap, conductivity, work function, and the structural evolution process before and after interface contact require interpretation via more advanced observation and analysis technology.
5. Difficulty in all‐printed electronics—the performance of printing electronic devices cannot be compared with that of silicon‐based electronic devices in a short time. In some of low‐cost disposal electronics, fully printing process is expected. Hence, it is necessary to explore fully solution‐processable semiconductors integrated with MXenes, such as semiconducting single‐walled carbon nanotubes, semiconducting conjugated small molecules, and to make them compatible with modern printing processes. In addition, all‐printed electronics based on MXene inks always suffered from device yield due to the oxidation issues of MXenes. The oxidative degradation compromises the intrinsic properties of the materials and increases the charge transfer barrier at the interface. Extra attempt was made to improve the environmental stability of MXenes by fine control of the quality of the parent MAX phase, chemical etching conditions, dispersion medium, storage conditions, defect passivation, edge selective functionalization, and development of polymer composites. Further investigation is needed to reveal the oxidative degradation mechanism and realize the deoxidizing of MXene.
6. Stability and reliability of the printed electronic device—since most MXenes and integrated device materials are susceptible to oxidation, the packaging technology is required to maintain bottom penetration and high barrier. The improvement of the life and environmental stability of electronic devices requires the selection of more advanced packaging technology. In addition to preventing physical damage, it is more imperative to prevent the erosion of trace amounts of water vapor, oxygen, and chemical components in the external environment, to prevent penetrating substances from reacting with functional materials, and to slow device aging. Furthermore, the wide distribution of electrical properties between devices remains a serious concern, primarily related to the limitations of the printing process, the uniformity of the thickness of the printed film layer, and the surface roughness that affects good contact between the interfaces.

## Conflict of Interest

The authors declare no conflict of interest.
